# A new perspective on data leakage prevention and adaptive attention in telecom churn prediction

**DOI:** 10.1038/s41598-026-49443-w

**Published:** 2026-04-26

**Authors:** Dang Tho Le, Manh Tuan Nguyen

**Affiliations:** 1 Institute of Innovation, University of Economics Ho Chi Minh City (UEH), Ho Chi Minh City, 700000 Vietnam; 2 School of Business Information Technology, University of Economics Ho Chi Minh City (UEH), Ho Chi Minh City, 700000 Vietnam

**Keywords:** Data leakage, Customer churn prediction, LOP-Net, Engineering, Mathematics and computing

## Abstract

Customer churn prediction is a critical task in business analytics, as inaccurate forecasts can lead to unnecessary marketing expenditure and suboptimal retention strategies. Despite substantial progress in machine learning and deep learning, two fundamental challenges remain under-addressed: data leakage introduced during preprocessing and resampling, and the lack of architectures capable of modeling multi-level nonlinear feature interactions. Leakage frequently arises when scaling or oversampling is applied before data splitting, allowing information from validation or test partitions to influence model training. Meanwhile, existing architectures often depend on conventional module stacking without introducing genuinely new mechanisms for capturing hierarchical dependencies. This study makes two primary contributions. First, we establish a rigorous, leakage-free experimental protocol featuring Auto Balance, a standalone adaptive oversampling algorithm that automatically searches for optimal sampling ratios while strictly isolating validation and test sets. Auto Balance mitigates common leakage pathways in imbalanced classification and provides statistically reliable performance estimation. Second, we propose LOP-Net, a novel deep learning architecture incorporating two new modules—Relationship-LSTM, which captures ordered and unordered relational patterns among features, and LOP-Attention, which models multi-level, nonlinear interaction structures beyond standard attention mechanisms. Comprehensive experiments across multiple telecom datasets demonstrate that the combination of the leakage-free pipeline and LOP-Net consistently outperforms strong baselines, achieving accuracy of up to 96% and Brier scores near 3%. Performance robustness is further supported by error-bar analysis across multiple random seeds, demonstrating stable variance behavior, and by paired t-tests comparing LOP-Net with alternative models, where all p-values fall below 5%, confirming statistically significant performance improvements. An integrated interpretability dashboard additionally supports practical churn analysis and strategic decision-making.

## Introduction

The telecommunications industry is facing increasing pressure to retain customers, as the cost of acquiring new users continues to rise amid fierce market competition. Customer churn prediction has therefore become a critical component for optimizing retention strategies and minimizing revenue loss. Telecom datasets are typically highly imbalanced, with churned customers forming a small fraction of the overall population. This imbalance poses a significant challenge for conventional machine learning models, such as Logistic Regression, Support Vector Machines, and XGBoost, which often struggle to maintain consistent predictive performance. In recent years, researchers have explored several strategies to improve churn prediction. Traditional ensemble methods, including hybrid bagging and stacking ensembles that integrate multiple gradient boosting models like LightGBM, CatBoost, and HistGBM, have achieved accuracies up to 96% while improving robustness against noisy or imbalanced data. However, these approaches often incur high computational costs and require careful hyperparameter tuning. Simultaneously, deep learning architectures such as BiLSTM–CNN and hybrid models incorporating Multi-Head Attention have demonstrated superior feature extraction and representation power, capturing complex sequential and contextual dependencies in telecom datasets. Furthermore, attention variants and relational LSTM models have been proposed to enhance adaptive relational learning, but many existing methods remain limited in their ability to flexibly adjust receptive fields or to explicitly model certain forms of high-order feature interaction. Another emerging trend is the use of data-centric preprocessing, including data transformation techniques (e.g., Z-score, Box-Cox, Weight-of-Evidence) and feature selection, which have been shown to significantly improve model performance by stabilizing feature distributions and emphasizing relevant attributes. These approaches complement architectural improvements, highlighting the importance of both effective preprocessing and advanced model design. To address the remaining challenges in churn prediction, we introduce two complementary contributions. First, we propose Auto Balance, an independent data-centric algorithm that automatically determines an effective resampling configuration without manual tuning. Second, we present LOP-Net, a deep learning architecture designed to capture complex feature interactions in conjunction with data balancing. LOP-Net integrates three key components Relationship-LSTM, LOP-Attention, and a multi-path representation block engineered to enhance relational expressiveness, adaptively focus on relevant feature interactions, and improve robustness. Experiments on multiple real-world telecom datasets demonstrate that, when combined with Auto Balance, LOP-Net achieves nearly 96% accuracy and an F1-score above 85%, while providing reliable probability estimates for identifying high-risk churners and implementing targeted retention strategies. Overall, this study highlights the importance of systematically combining leakage-aware preprocessing with adaptive deep learning architectures in churn prediction.

## Related work

### Literature review

The customer churn problem in the telecommunications industry remains a major challenge and a long-standing interest for machine learning researchers. Early studies demonstrated that classical models such as Support Vector Machines^1^, Logistic Regression^2^, K-Nearest Neighbors^3^, and Decision Trees^4,5^ could achieve promising results, with accuracy approaching 99%, by leveraging hyperplane separation or tree-based heuristics. And a recent study in Frontiers in Artificial Intelligence developed a predictive analytics model for telecom customer churn that integrates advanced preprocessing, feature selection, and multiple machine learning classifiers, reporting strong performance particularly with Support Vector Machines^6^. This work highlights the importance of preprocessing and interpretability when applying ML models to telecom churn data. In parallel, ensemble methods like Random Forest^7^ and XGBoost^8^ improved sensitivity metrics such as Recall and Precision, achieving up to 90%. Ahmad et al^9^ developed a customer churn prediction framework for telecom using big data platforms. They combined statistical features from call records with Social Network Analysis (SNA) features, capturing relational information between customers. Implemented on Apache Spark over Hadoop, their XGBoost model achieved an AUC of 93.3%, showing that integrating social network features with scalable big data processing can significantly improve churn prediction accuracy. This complements other approaches using ensemble methods, deep learning, and feature preprocessing for telecom churn prediction. While lightweight and fast, these models struggle with noisy or imbalanced datasets. Abdelhady and Mohamed^10^ proposed an AI-driven churn prediction framework for telecom using a Random Forest classifier with SMOTE and class weighting. The model achieved 95% accuracy and AUC 0.89, highlighting key usage and service interaction features. This study demonstrates how AI can support actionable customer retention strategies within CRM systems. Ensemble learning emerged to address these limitations. Hybrid bagging models^11^ and frameworks such as XAI-Churn TriBoost^12^ demonstrated notable improvements in prediction robustness, reaching F1-scores above 90%. Stacking ensembles integrating gradient boosting models—LightGBM, CatBoost, HistGBM—achieved accuracies up to 96.39%^13^. However, these approaches incur high computational cost and require careful hyperparameter tuning, limiting scalability. In addition, Faraji Googerdchi et al^14^ proposed multi-objective evolutionary ensemble classifiers (MOEECs) for telecom churn prediction, combining clustering with evolutionary optimization to maximize both accuracy and diversity among base classifiers. Their MOEEC models achieved up to 97.30% accuracy and AUC 93.76%, outperforming traditional and other ensemble methods. However, these approaches incur high computational cost, rely on careful clustering and hyperparameter tuning, and offer limited interpretability, which may hinder practical deployment in large-scale or operational telecom environments. Recent research has increasingly focused on deep learning-based architectures, capable of extracting richer feature representations with less manual parameter tuning. For example, BiLSTM–CNN models^15^ effectively capture sequential dependencies while highlighting salient features via convolutional layers. Hybrid architectures such as CCP-Net^16^ integrate Multi-Head Attention and BiLSTM to enhance representational power, demonstrating the evolution from traditional statistical models to advanced deep learning methods for churn prediction.

### Research gap and motivation

Despite these advances, existing studies face several limitations that hinder applicability in real-world telecom environments. First, data imbalance remains a major challenge. Common strategies like oversampling and undersampling are often insufficiently documented, raising reproducibility concerns. More critically, some studies^17–20^ perform data balancing before dataset splitting, introducing data leakage and leading to overly optimistic evaluations. Even rigorously designed studies^8^ face computational overhead due to repeated splitting and retraining for optimal balancing, motivating the need for automated, efficient solutions. Second, at the architectural level, most models have not fully exploited adaptive mechanisms tailored to task-specific structures. While methods such as Dynamic Convolution^21^ and Dynamic Head^22^ introduce dynamic behavior in CNNs, they operate in spatial feature spaces, unlike attention heads in Transformer architectures which model contextual dependencies. Existing approaches fail to dynamically adjust the receptive field of attention heads, limiting performance on sequential and tabular data with complex local and global interactions. Third, LSTM variants aiming to enhance relational learning still have limitations. For instance, Relation-Gated LSTM^23^ regulates hidden states without affecting cell state updates; Context-Gate LSTM^24^ balances context and target only within decoders; Relational Memory Core^25^ applies self-attention among memory slots rather than gating within cells; Coupled-LSTM^26^ focuses on parallel sequence interaction rather than intra-sequence dependencies. Finally, few studies have demonstrated practical deployment of churn prediction models in real-world telecom environments, underscoring the need for approaches that are both theoretically robust and operationally applicable. To address these gaps, we introduce **Auto Balance**, an independent data-centric algorithm for automatic, leakage-free resampling, and **LOP-Net**, a deep learning architecture that integrates Relationship-LSTM, LOP-Attention, and a multi-path representation block. This design enables the model to capture high-order feature interactions, adaptively focus on relevant dependencies, and enhance robustness against data imbalance, addressing commonly observed limitations in prior churn prediction studies.

The proposed LOP-Net adopts a hierarchical modeling strategy to explicitly disentangle structural and relational dependencies in customer behavior data. The first component, the modified Transformer with LOP-Attention, is designed to capture intra-group interactions among features within each temporal snapshot. By combining self-attention with adaptive convolutional receptive fields, this module focuses on learning multi-scale structural patterns inside feature groups, such as correlations among service usage attributes or financial indicators at a given time step. The second component, the Relationship LSTM, operates on the representations produced by the Transformer encoder and models the inter-group relational evolution across time. Its additional relationship gate allows the network to selectively preserve or update memory based on changing interactions between consecutive feature groups, thus emphasizing long-term relational dynamics rather than isolated temporal signals. These two modules play complementary roles: while the Transformer-based encoder extracts rich local and multi-scale structural representations within each group, the Relationship LSTM integrates these representations over time to capture global relational trajectories. This hierarchical separation enables the model to handle both structural heterogeneity and temporal dependency in a unified framework, which is particularly suitable for customer churn prediction where both short-term behavior patterns and long-term relationship trends are critical.

- **Auto Balance:** This study introduces an automated data balancing mechanism that adaptively determines the sampling ratio, oversampling method, and resampling seed through internal validation, rather than relying on manually tuned hyperparameters as in prior work. All operations are conducted strictly within the training split, ensuring that the validation and test sets remain completely isolated and that no form of train–test leakage can occur. The mechanism directly optimizes the F1-score—an essential metric for imbalanced classification—using a binary-search–based heuristic to efficiently explore the sampling-ratio space. In contrast to traditional SMOTE-based approaches with fixed configurations, the Auto Balance controller evaluates multiple resampling strategies and selects the combination that yields the strong validation performance. This adaptive design enables the system to adjust automatically to datasets with different imbalance severities, a capability that has been largely underexplored in the churn prediction literature.
- **Relationship LSTM**: Distinct from prior relational extensions of LSTM, the Relationship LSTM proposed in this study embeds a relationship gate $r_{t}$ directly into the cell state update equation. This gate controls the degree of interaction between old and new information by dynamically weighting the influence of the previous memory cell $c_{t-1}$ and the current input $x_{t-1}$. The gate computation is derived from both the current input and a normalized projection of the prior memory state, allowing the model to learn contextual dependencies between consecutive memory states in a self-regulated manner. This internal gating mechanism differs from earlier works in that relational modulation is applied directly within the cell state update, rather than only at the output or attention level, where relational gates were applied only to output layers or attention spaces and thus failed to explicitly capture intra-step feature group interactions. Combined with the forward–backward (bidirectional) structure, Relationship LSTM preserves the long-term memory capability of conventional LSTM while effectively modeling the cross-feature relationships within each timestep — a crucial property for churn prediction, where behavioral, payment, and complaint features often exert joint and simultaneous influence.
- **LOP-Attention**: An architectural-level extension of the Transformer designed to mitigate structural homogeneity among attention heads in conventional self-attention mechanisms. Instead of assuming that all heads operate under a fixed and shared receptive structure, LOP-Attention enables each attention head to *self-modulate its structural representation* through a dedicated one-dimensional convolution applied at the post-attention stage. After the standard scaled dot-product attention computation:
  1 $$\begin{aligned} O_h = \operatorname {Softmax}\!\left( \frac{Q_h K_h^{\top }}{\sqrt{d_h}}\right) V_h, \end{aligned}$$
  each head *h* is equipped with a learnable continuous receptive field parameter that controls the effective span of a head-specific convolutional kernel. Rather than selecting discrete kernel sizes, LOP-Attention parameterizes the receptive field using a differentiable radius variable $r_h$, which is optimized jointly with the attention weights during training. A Gaussian soft mask is then constructed to modulate a shared maximum-size convolution kernel, yielding a smooth and adaptive receptive field for each head:
  2 $$\begin{aligned} O_h^{\text {conv}} = \operatorname {Conv1D}\bigl (O_h,\, W_h^{\text {conv}} \odot m_h(r_h)\bigr ). \end{aligned}$$
  This design introduces an additional learning dimension within the Transformer architecture, where both attention weights and head-specific structural extents are jointly optimized in an end-to-end manner. As a result, different heads naturally specialize in capturing local or long-range dependencies, forming a unified multi-scale representational manifold within a single Transformer block. Unlike prior approaches such as Dynamic Convolution or Dynamic Head mechanisms, which adapt kernel weights or channel configurations within convolutional feature spaces, LOP-Attention directly modulates the geometric structure of self-attention at the per-head level. Consequently, LOP-Attention extends the Transformer from a purely weight-learning model to a structure-learning architecture, enabling localized adaptability without sacrificing global dependency modeling.
- **Practical Application:** The proposed LOP-Net has been deployed and tested in a real-world telecom customer management platform, where it supports the automatic identification and classification of customers according to their churn risk levels. This mechanism introduces an additional degree of adaptability within the Transformer architecture by allowing each attention head to learn a head-specific receptive field. As a result, different heads can operate at different effective scales, enabling multi-scale representation learning within a single attention block. Unlike Dynamic Convolution or Dynamic Head, which operate in the CNN feature space, LOP-Attention applies adaptive convolution directly to attention head outputs, preserving global dependency modeling while enhancing localized adaptability.

## Methodology

### Experimental customer churn

**Fig. 1:**
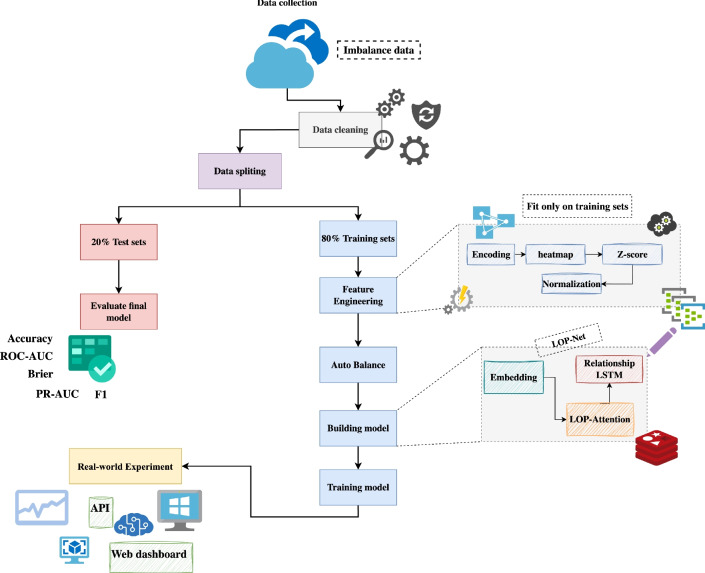
Workflow of experiment.

The experimental workflow, illustrated in the Fig. 1 below, consists of six main stages: (1) data collection, (2) data cleaning, (3) data splitting of the imbalanced dataset into 80% training and 20% testing sets, (4) Feature engineering, (5) Model construction, (6) Experimental evaluation, and (7) practical deployment.

### Data collection

To help the model achieve high generalization, we performed on 2 Telecom datasets. The data is taken from Kaggle^27^, UC Irvine^28^ is completely open access. Table 1 below shows the percentage distribution between churn Churn and Non Churn.

**Table 1:** Distribution of churn and non-churn samples across telecom datasets.

Dataset	Churn (%)	Non-Churn (%)
Orange Telecom	14.29	85.71
Iranian Telecom	15.72	84.28

### Data cleaning

At this stage, the raw data are cleaned to remove noisy and invalid elements. Specifically, duplicate records, missing values (NaN), and formatting errors are completely eliminated rather than being imputed or replaced. This process is solely intended to ensure the integrity of the input data and does not involve any statistical computation or parameter learning from the dataset; therefore, it does not introduce any data leakage.

### Data spliting

After data cleaning, the dataset is divided such that 80% is used for training and 20% for testing, denoted as $D_{80\% \text {training sets}}$ and $D_{20\% \text {test sets}}$, respectively. Both the training and testing sets remain imbalanced. The Telecom Churn dataset used in this study is a static tabular dataset where each row corresponds to an individual customer at a single point in time. Nevertheless, we ensured that all preprocessing steps, such as feature encoding, scaling, and resampling, were fitted exclusively on the training set to avoid data leakage between training and testing phases. At this stage, all data transformations are applied exclusively to the training set, ensuring a strictly controlled experimental procedure.

### Label encoding

After dividing the dataset into two independent subsets (training and testing), categorical features are converted into numerical form using the Label Encoding technique. This encoding process follows the “fit–transform” principle on the training set and applies only the “transform” operation on the testing set, ensuring that the encoder does not learn any information from the test data. This approach completely eliminates the risk of data leakage during preprocessing and maintains reproducibility throughout the training and evaluation phases.

### Feature selection

**Fig. 2:**
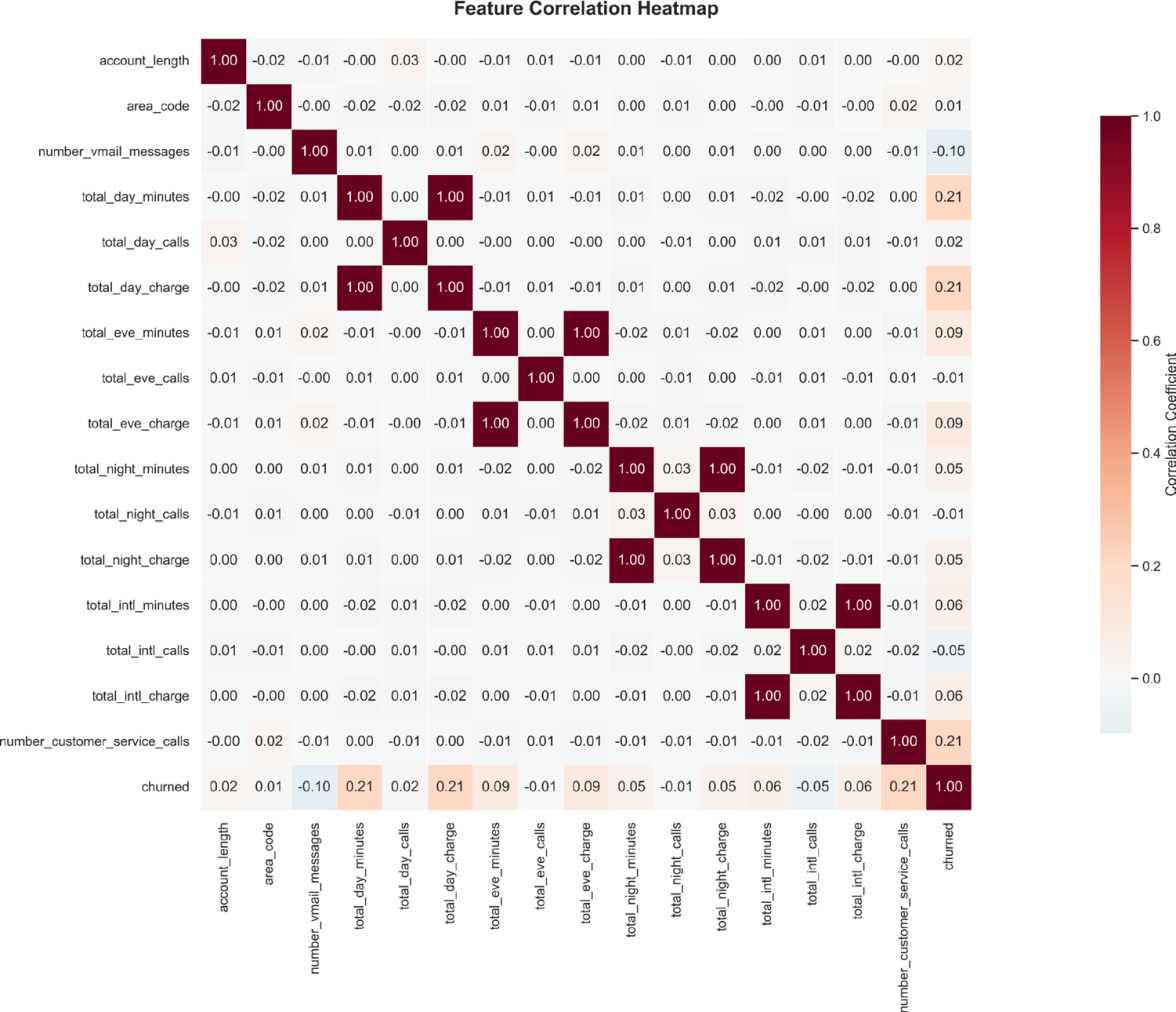
Hetmap of orange telecom.

**Fig. 3:**
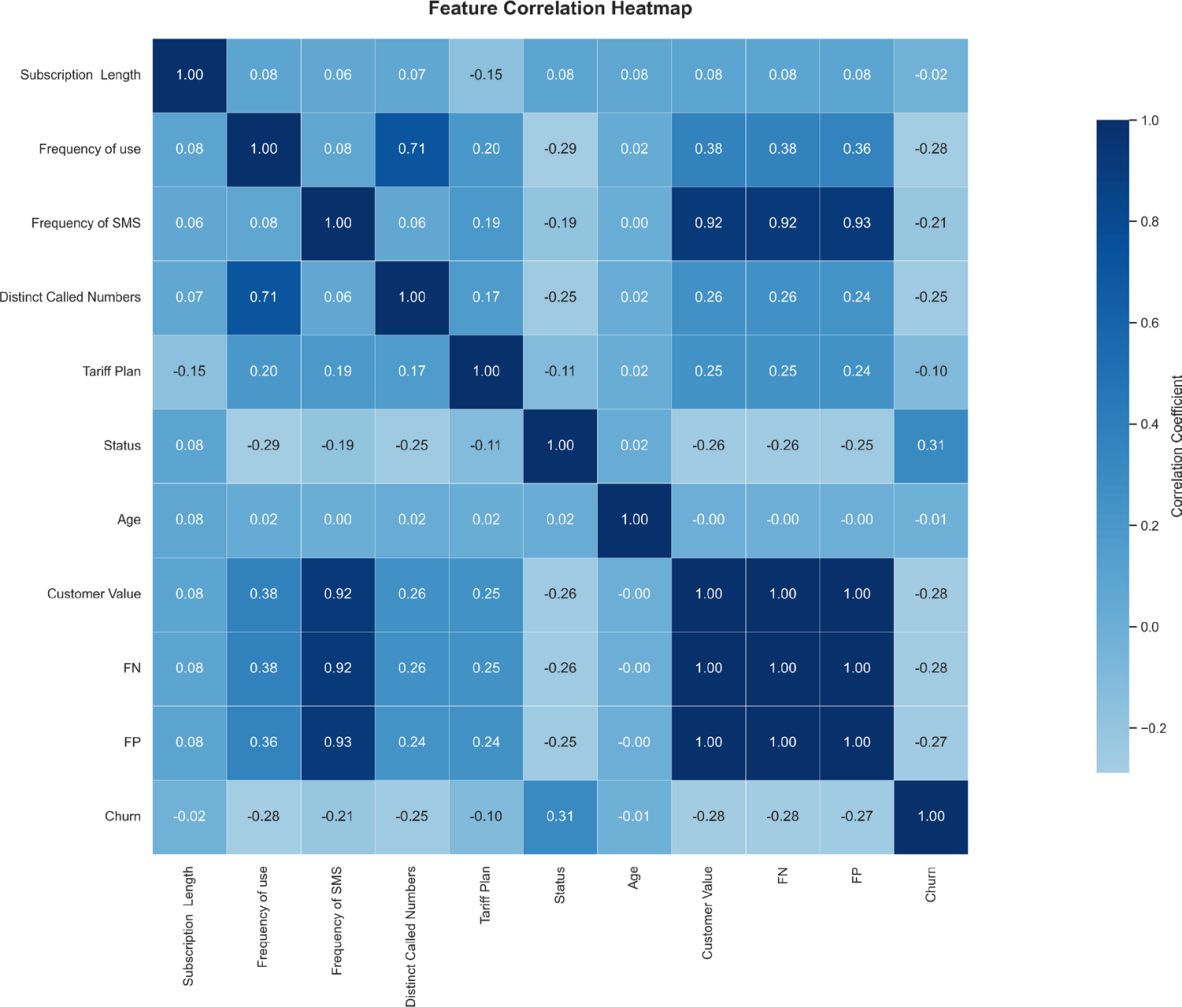
Hetmap of Iranian telecom.

Since our model is carefully designed and suitable for learning various nonlinear features, we only remove the features that are truly unnecessary, while retaining those with weak effects. Partly, keeping features with high nonlinearity makes the data more complex, thereby allowing a high-quality model to demonstrate its stability and performance. The remaining part, removing unnecessary features, is determined by the heatmap correlation. The heatmap correlation is computed entirely on the training set to prevent information leakage from the test set during the feature selection process. For example, in the Orange dataset, features such as ‘state’, ‘account_length’, ‘area_code’, and ‘phone_number’ are removed since they do not affect the churn problem. Additionally, these features typically do not have a large impact on Churn, as these features are identifiers, like ’phone_number’ which cannot necessarily determine what percentage of customers will leave or stay. Figure 2 and Fig. 3 show heatmap correlation on both telecom datasets.

### Data balance

Unlike traditional resampling techniques such as SMOTE^29^, ADASYN^30^, or Random Oversampling^31^, which are typically applied in a fixed manner and may inadvertently introduce data leakage by manipulating the entire training set at once, Auto Balance is designed as an adaptive and fully leakage-free resampling algorithm. Specifically, Auto Balance considers a set of candidate generators.

$$\begin{aligned} S \in \{\text {RANDOM}, \text {SMOTE}, \text {ADASYN}, \text {SMOTEENN}\} \end{aligned}$$

and for each generator searches for an optimal sampling ratio $\alpha ^{*}$ that maximizes validation performance. To estimate the effectiveness of each resampling configuration, the module employs a lightweight proxy classifier (a small MLP) and evaluates the F1-score on an internal validation split. Importantly, all operations—normalization, resampling, and data splitting—are performed strictly within the training portion of the data, ensuring a completely leakage-free procedure and full reproducibility. The validation subset is never affected by oversampling, preserving the statistical independence necessary for unbiased performance estimation.

To improve search efficiency, Auto Balance first performs a dynamic binary search over the sampling-ratio space for generators that support continuous ratios (e.g., Random Oversampling, SMOTE, ADASYN). However, because certain datasets may exhibit non-monotonic behaviour or unstable F1-score regions, the module also integrates Bayesian Optimization as a fallback mechanism. When the post-resampling class distribution deviates excessively from the target balance (e.g., the minority-to-majority ratio changes by more than 20%), Auto Balance automatically switches to a Gaussian Process–based Bayesian optimizer to refine $\alpha ^{*}$ in a statistically principled manner. This hybrid strategy combining binary search and Bayesian Optimization ensures both efficiency and robustness, outperforming purely heuristic or exhaustive search approaches.

Class imbalance is therefore handled through a fully automatic, leakage-free resampling framework designed to enforce methodological rigour and strict statistical independence across training, validation, and test partitions. Naively applying oversampling methods (e.g., SMOTE or ADASYN) often contaminates validation or test sets because synthetic samples may inadvertently encode distributional information from held-out data. To prevent this, we employ a rigorous evaluation protocol in which oversampling is applied *only* inside the inner-training split, while all validation subsets remain untouched.

Given the outer training set $(X_{\textrm{train}}, y_{\textrm{train}})$, the procedure evaluates each generator *S* (Random Oversampling, SMOTE, ADASYN, and SMOTE–ENN) in combination with a sampling ratio $\alpha$. For each configuration $(S, \alpha )$, the outer-training set is partitioned into an inner-training and inner-validation subset via stratified sampling. A feature-normalization transformation is then fitted *exclusively* on the inner-training data and subsequently applied to both subsets, ensuring that normalization parameters never incorporate information from any validation or test instance.

Oversampling is then applied only to the normalized inner-training subset via $S(\alpha )$, while the inner-validation subset remains unchanged. The light deep learning classifier (one hidden layer) is trained on the resampled inner-training data and evaluated on the untouched inner-validation data. The resulting validation F1-score serves as the objective function for selecting the optimal resampling configuration. Because the validation split contains no synthetic samples and no parameters trained on it propagate back into normalization or sampling, the resulting estimate is unbiased.

For generators requiring a continuous ratio (e.g., SMOTE or ADASYN), we restrict the search to

$$\begin{aligned} \alpha \in [0.7, 1.0]. \end{aligned}$$

If the resulting distribution after resampling remains substantially imbalanced (e.g., minority/majority $<0.8$), we further refine $\alpha$ using Bayesian Optimization. This additional step stabilizes the choice of $\alpha$ when its effect on validation F1-score is non-monotonic or contains flat regions. Crucially, Bayesian Optimization evaluates all candidate values of $\alpha$ using the same leakage-free inner-splitting protocol, ensuring that no adaptive mechanism ever exploits validation data improperly. As a result, both the initial search and the Bayesian refinement preserve strict safeguards against data leakage and model-selection bias.

We acknowledge that relying solely on binary search over the sampling ratio $\alpha$ may overlook global extrema, because the mapping between the oversampling ratio and model performance,

3 $$\begin{aligned} \alpha \,\longmapsto \, \textrm{F1}(\alpha ), \end{aligned}$$

is neither monotonic nor unimodal in practical churn prediction tasks. This irregularity arises from complex interactions among synthetic sample quality, distributional shifts induced by oversampling, classifier sensitivity, and high-dimensional feature representations. Consequently, interval-halving may converge to suboptimal regions of the search space. To address this limitation, Auto Balance integrates a second-stage optimization procedure based on *Bayesian Optimization* (BO), which provides an efficient framework for optimizing expensive and derivative-free black-box functions such as the inner-validation F1-score. The optimization objective is formally defined as:

4 $$\begin{aligned} \alpha ^{*} = \arg \max _{\alpha \in [\alpha _{\min },\, \alpha _{\max }]} \, \mathscr {F}(\alpha ), \end{aligned}$$

where $\mathscr {F}(\alpha )$ denotes the validation F1-score obtained from the leakage-free training procedure under sampling ratio $\alpha$. Bayesian Optimization models the unknown objective function using a Gaussian Process (GP):

5 $$\begin{aligned} \mathscr {F}(\alpha ) \sim \mathscr{G}\mathscr{P}\!\left( m(\alpha ),\, k(\alpha , \alpha ')\right) , \end{aligned}$$

where $m(\alpha )$ is the prior mean function and $k(\alpha ,\alpha ')$ is a covariance kernel that imposes smoothness assumptions over the F1 landscape. At iteration *t*, BO selects the next candidate ratio $\alpha _t$ by maximizing an acquisition function $\mathscr {A}(\alpha )$:

6 $$\begin{aligned} \alpha _t = \arg \max _{\alpha }\, \mathscr {A}(\alpha ), \end{aligned}$$

where Expected Improvement (EI) is a commonly used acquisition strategy:

7 $$\begin{aligned} \mathscr {A}_{\textrm{EI}}(\alpha ) = \mathbb {E}\!\left[ \max \bigl (0,\, \mathscr {F}(\alpha ) - \mathscr {F}(\alpha ^{+})\bigr ) \right] , \end{aligned}$$

and $\alpha ^{+}$ denotes the best-performing sampling ratio found so far. The new observation $\mathscr {F}(\alpha _t)$ is then used to update the GP posterior, gradually refining the surrogate model of the F1 landscape. In Auto Balance, Bayesian Optimization is invoked only when the binary-search ratio produces a post-resampling imbalance that violates a safety criterion (e.g., minority-to-majority ratio below a threshold). This creates a hybrid optimization strategy:

- Binary search offers a fast coarse estimate of a promising sampling ratio.
- Bayesian Optimization ensures that the final ratio $\alpha ^{*}$ is globally competitive even when $\mathscr {F}(\alpha )$ is irregular, noisy, or multimodal.

**Fig. 4:**
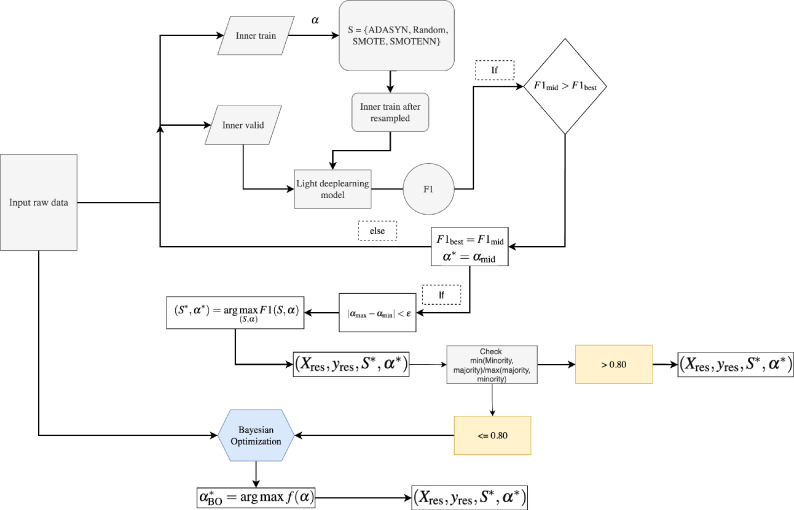
Workflow of auto balance.

Through this two-stage framework, Auto Balance provides an adaptive, leakage-free, and performance-driven mechanism for handling severe class imbalance, offering substantial advantages over traditional static resampling techniques in customer churn prediction. The overall workflow of Auto Balance is presented in Fig. 4, and the corresponding pseudocode is provided below.

Once the optimal configuration $(S^*, \alpha ^*)$ has been identified, the selected sampler is applied *exactly once* to the full outer–training set (before any model fitting). This final resampling is performed directly on the raw, unstandardized features, after which a new scaler is fitted solely on the resampled training data prior to downstream training. Importantly, the outer–validation and test sets remain completely untouched throughout the entire process. This design preserves distributional independence, prevents synthetic sample leakage, ensures reproducibility, and aligns with the principles of nested cross-validation, while maintaining computational efficiency suitable for deep learning pipelines.

Overall, the procedure yields a principled, stable, and fully leakage-free method for selecting both the oversampling strategy and its sampling ratio. It avoids the two most critical pitfalls in imbalance handling: (i) contamination of validation or test sets with synthetic information, and (ii) artificially inflated performance estimates resulting from performing resampling or scaling before data splitting. By integrating strict data isolation with adaptive ratio refinement via Bayesian search, Auto Balance ensures that the final resampled training distribution is statistically sound and empirically optimal for subsequent model learning.

**Algorithm 1:**
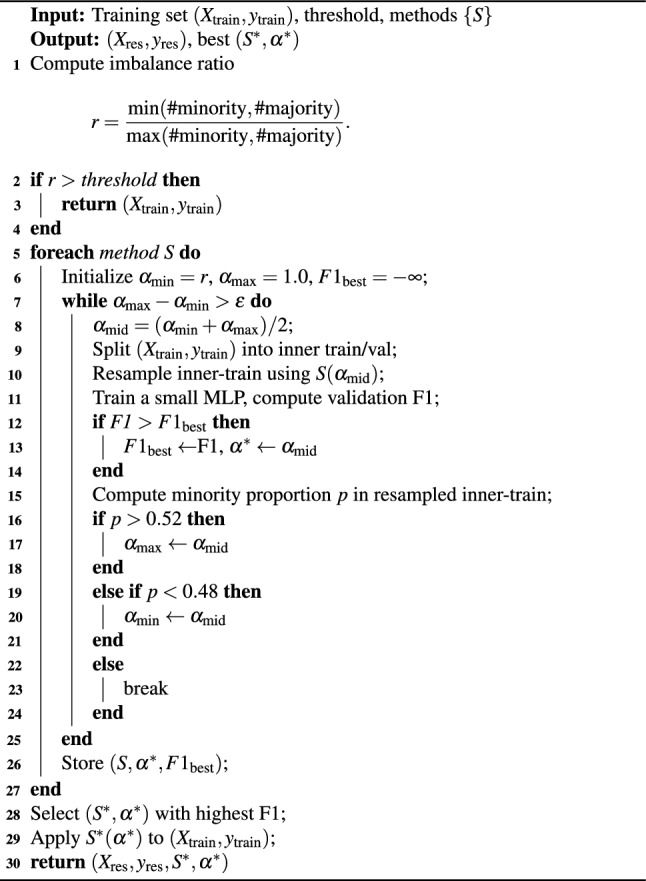
Auto-Balance (Leakage-Free Binary Search).

### Model architecture

The proposed model consists of several sequential blocks, including Embedding, LOP-Attention, and Relationship LSTM. LOP-Net is designed as a hierarchical architecture that separates structural feature modeling from temporal relational modeling. The modified Transformer encoder with LOP-Attention first captures intra-group interactions within each feature group by learning multi-scale dependencies through self-attention and adaptive convolutional kernels. The resulting representations are then passed to the Relationship LSTM, which focuses on modeling inter-group relational evolution over time using an additional relationship gate to selectively preserve and update long-term dependencies. These two components play complementary roles: the Transformer extracts rich structural patterns at each time step, while the Relationship LSTM integrates them across time to characterize global behavioral trajectories. This hierarchical design enables LOP-Net to jointly capture local feature interactions and long-term relational dynamics in a unified framework.

#### Embedding

Embedding is a method for representing objects as vectors in a high-dimensional space. The use of an embedding layer in the model plays a crucial role in extracting and compressing customer feature information. Through a linear transformation from the input space to a higher-dimensional latent space, this process allows for richer representation of data in the feature space, thereby enhancing the predictive performance for customer churn. Consequently, the ability to expand the dimensionality enables the model to capture nonlinear relationships and latent interactions among the input features.

#### LOP-attention

LOP-Attention is implemented directly within the Transformer architecture, where each attention head is equipped with an adaptive and continuous temporal receptive field. After computing the scaled dot-product self-attention, each head produces an attention-refined representation $O_h$. Instead of applying a shared or fixed convolution, LOP-Attention performs a head-wise 1D convolution along the temporal dimension of each $O_h$ independently. The effective receptive field of each head is controlled by a learnable continuous radius parameter, rather than a discrete kernel size. A Gaussian soft mask is applied to a maximal convolution kernel, yielding a smooth and differentiable weighting over temporal positions. This design enables each attention head to adapt its temporal focus during training, allowing different heads to specialize in short-range or long-range dependency modeling. Unlike prior convolutional attention variants that apply convolution after concatenating head outputs or directly on input feature maps, LOP-Attention applies convolution before head aggregation and after attention refinement. This preserves head-specific representations while enhancing them with multi-scale temporal context. Importantly, the convolution operates in the attention-refined feature space, enabling effective integration of local and global information without altering the core Transformer attention mechanism. To ensure stable sequence alignment and reduce boundary distortions when receptive fields vary across heads, we further introduce a smart padding strategy based on edge replication. This maintains consistent sequence length and improves gradient stability during training. To the best of our knowledge, this is the first work in churn prediction to introduce per-head, continuously adaptive receptive fields at the attention output level. The proposed design opens a new direction for multi-scale adaptive attention learning within Transformer-based architectures.

- **Multi-Head Self-Attention Formulation:** Let $X \in \mathbb {R}^{B \times T \times D}$ denote the input sequence. The input is linearly projected into Query (*Q*), Key (*K*), and Value (*V*) spaces as Equation (6):
  6 $$\begin{aligned} Q = XW^Q,\quad K = XW^K,\quad V = XW^V, \end{aligned}$$
  where $W^Q, W^K, W^V \in \mathbb {R}^{D \times D}$. For each attention head $h = 1,\dots ,H$, self-attention is computed by Equation (7):
  7 $$\begin{aligned} O_h = \textrm{softmax} \left( \frac{Q_h K_h^\top }{\sqrt{d_h}} \right) V_h, \quad d_h = \frac{D}{H}. \end{aligned}$$
- **Continuous Receptive-Field Parameterization:** To enable adaptive temporal modeling, each attention head *h* is associated with a learnable continuous radius parameter. We define a mapping as Equation (8):
  8 $$\begin{aligned} \phi : \mathbb {R}^H \rightarrow [0, r_{\max }]^H, \qquad r_h = \phi (\alpha _h, s_h) = \textrm{clip}(\alpha _h \cdot s_h, 0, r_{\max }), \end{aligned}$$
  where $\alpha _h$ and $s_h$ are trainable parameters and $r_{\max } = \lfloor k_{\max }/2 \rfloor$ denotes the maximum allowable radius. The mapping $\phi$ is continuous and almost everywhere differentiable, ensuring stable gradient propagation during training.
- **Gaussian Soft Mask as a Continuous Relaxation:** Instead of discretely selecting kernel sizes, we introduce a smooth weighting over kernel positions. Let $m \in \{0,\dots ,k_{\max }-1\}$ index positions and let $c = \lfloor k_{\max }/2 \rfloor$ denote the kernel center. For each head *h*, we define a Gaussian soft mask as Equation (9) :
  9 $$\begin{aligned} M_h(m) = \exp \!\left( -\frac{(|m - c| - r_h)^2}{2\sigma ^2} \right) , \end{aligned}$$
  where $\sigma> 0$ controls the smoothness of the mask. This formulation provides a continuous relaxation of discrete kernel selection while preserving locality-aware inductive bias.
- **Head-wise Convolution with Adaptive Receptive Fields:** Let $W_h^{\textrm{conv}} \in \mathbb {R}^{d_h \times d_h \times k_{\max }}$ denote the maximal convolution kernel of head *h*. The effective kernel is defined as Equation (10):
  10 $$\begin{aligned} W_h^{\textrm{eff}}(m) = W_h^{\textrm{conv}}(m) \odot M_h(m). \end{aligned}$$
  Given the padded attention output $O_h^{\textrm{padded}}$, the head-wise temporal convolution is computed as Equation (11):
  11 $$\begin{aligned} O_h^{\textrm{conv}} = \textrm{Conv1D} \big ( O_h^{\textrm{padded}}, W_h^{\textrm{eff}}, b_h \big ) \in \mathbb {R}^{B \times T \times d_h}. \end{aligned}$$
- **Head Aggregation and Residual Learning:** All convolved head outputs are concatenated and projected back to the model dimension as Equation (12):
  12 $$\begin{aligned} \text {Output} = \textrm{Concat} \big [ O_1^{\textrm{conv}}, O_2^{\textrm{conv}}, \dots , O_H^{\textrm{conv}} \big ], \end{aligned}$$
  followed by a linear projection, residual connection, and layer normalization as Equation (13):
  13 $$\begin{aligned} Y = \textrm{LayerNorm}(X + \text {Output} W_O + b_O). \end{aligned}$$

**Fig. 5:**
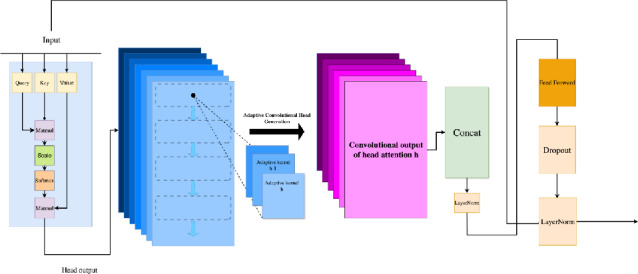
Structure of LOP-Attention.

To highlight the contributions of LOP-Attention relative to prior dynamic methods, Table 2 summarizes the key differences between Dynamic Convolution^21^, DyHead^22^, and the proposed method. The LOP-Attention block is illustrated by Fig. 5 below, and is described by the following pseudo code.

**Table 2:** Comparison of LOP-Attention with existing dynamic convolution mechanisms.

Method	Domain of Operation	Adaptation Target	Granularity	Transformer	Receptive Field Type
Dynamic Convolution^21^	CNN feature maps	Kernel weights	Global	✗	Static
Dynamic Head^22^	CNN detection heads	Kernel weights	Per-head	✗	Static
LOP-Attention	Attention embeddings	Receptive field radius	Per-head	✓	Learnable continuous

**Algorithm 2:**
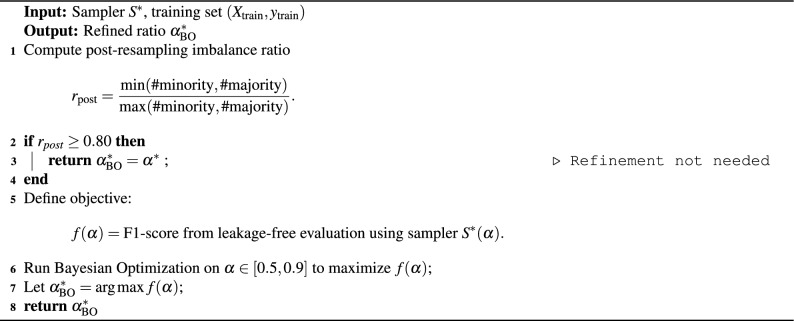
Bayesian Optimization for Sampling Ratio Refinement.

#### Relationship LSTM

Although LOP-Attention is powerful in learning representations from local to global contexts, this attention mechanism still lacks the capability to remember and model long-term dependencies across time steps. To address this limitation, we propose the Relationship LSTM, a bidirectional variant of LSTM extended with a *relation gate* $r_t$. Unlike the traditional LSTM with gates $f_t, i_t, o_t$, the $r_t$ gate regulates the interaction between the current memory cell and the previous one, enabling the model to dynamically learn state connections and capture changes in customer behavior over time.

- **Relation Gate** $r_t$. Given the previous memory state $c_{t-1}$, we first normalize and project it into a new space as Equation (16):
  16 $$\begin{aligned} y_{t-1} = \tanh (\text {LayerNorm}(c_{t-1})) \end{aligned}$$
  The relation gate $r_t$ is then generated via as Equation (17):
  17 $$\begin{aligned} \begin{aligned} r_t^{\text {raw}} = W_r x_t + U_r h_{t-1} + b_r\\ r_t = \sigma \big (\text {LayerNorm}(r_t^{\text {raw}} + W_y y_{t-1})\big ) \end{aligned} \end{aligned}$$
  where $W_y \in \mathbb {R}^{d_h \times d_h}$ is a learnable projection matrix that maps the context from the previous memory cell into the current gating space.
- **Memory Cell Update.** The memory update is extended as follows Equation (18):
  18 $$\begin{aligned} c_t = (f_t + r_t \odot (1 - f_t)) \odot c_{t-1} + (1 - r_t) \odot (i_t \odot \hat{c}_t) \end{aligned}$$
  allowing a flexible combination of long-term and short-term information. This mechanism enables the model to preserve important past features while quickly adapting to new changes, thus improving the representation of complex sequential relationships in customer churn behavior.
- **Bidirectional Extension.** In addition to the forward pass, Relationship LSTM is designed to exploit bidirectional context. Specifically, besides the forward branch processing the sequence in the natural order ($x_1 \rightarrow x_T$), a backward branch is trained in parallel to capture reverse temporal information ($x_T \rightarrow x_1$). At each time step *t*, the outputs from both directions are concatenated as Equation (19):
  19 $$\begin{aligned} h_t = [h_t^f, h_t^b] \end{aligned}$$

**Fig. 6:**
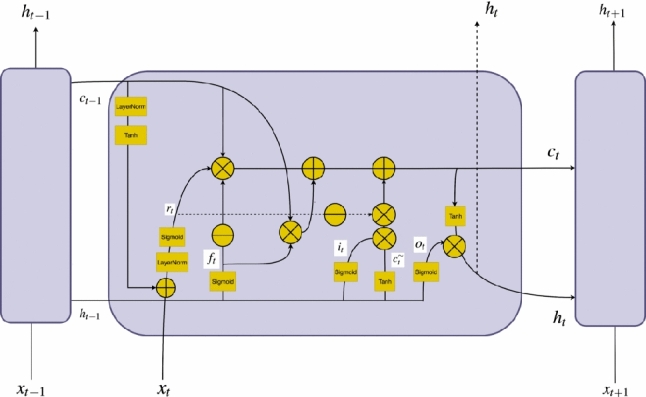
Structure of relationship LSTM.

**Fig. 7:**
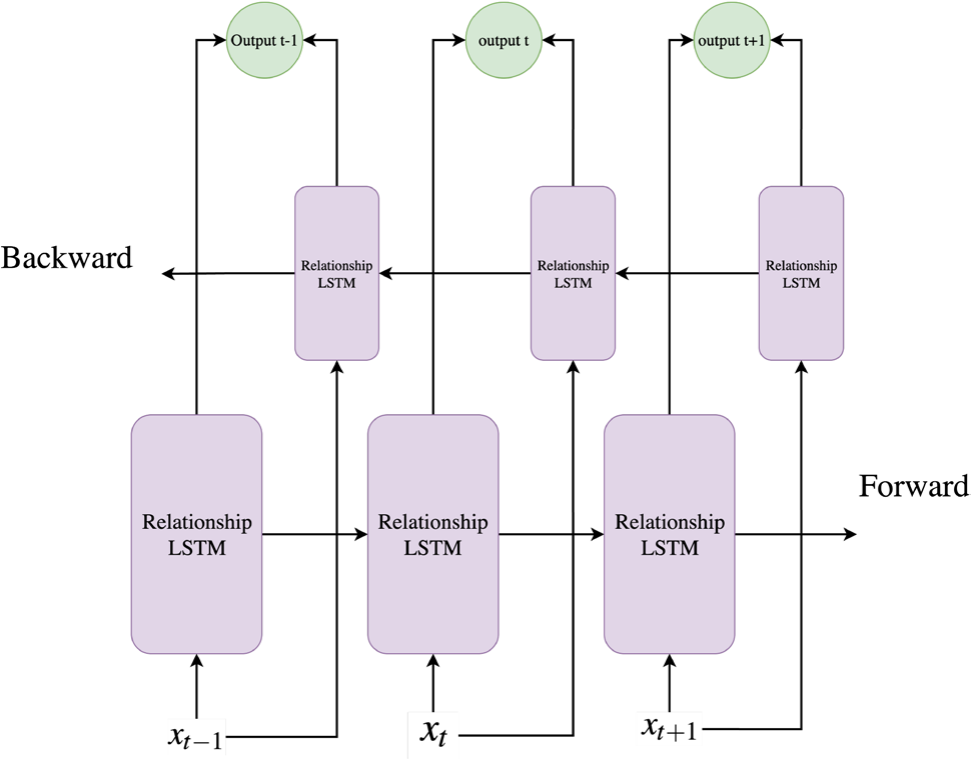
Workflow of bidirectional relationship LSTM.

The combination allows the model not only to understand the temporal progression of relationships but also to recognize the backward influence of future events (e.g., impending churn behavior) on earlier features. As a result, the Relationship LSTM is capable of capturing bidirectional contextual dependencies and modeling customer behavior sequences with higher semantic depth, which is particularly useful in Customer Churn Prediction, where churn signals are often non-linear and exhibit backward-propagating effects over time. To the best of our knowledge, no prior work has integrated the dynamic relation gate $r_t$ in LSTM for customer churn prediction, nor in tabular sequential models in general. The structure of Relationship LSTM can be shown as below Fig. 6. And the bidirectional Relationship can be shown as below Fig. 7. In Fig. 6, different symbols are used to denote basic mathematical operations within the Relationship LSTM architecture. The symbol $\oplus$ represents element-wise addition between two signals. The symbol $\otimes$ denotes element-wise multiplication, which is mainly used to implement gating mechanisms for controlling information flow. The symbol $\ominus$ indicates a complementary transformation, where an input value *x* is mapped to its complement $1 - x$. This operation is applied to model inhibitory effects and to regulate the contribution of retained and newly injected information in the memory cell.

**Fig. 8:**
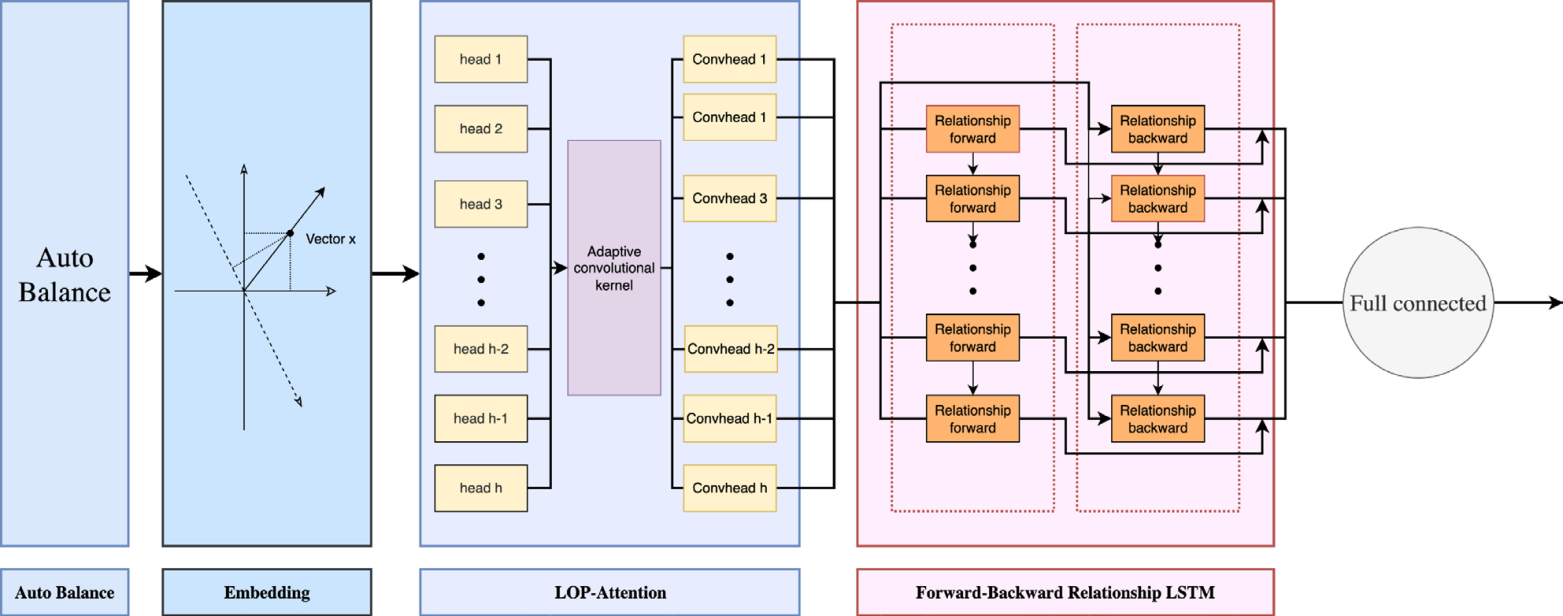
Structure of LOP-Net.

The structure of LOP-Net can be shown as below Fig. 8.

**Algorithm 3:**
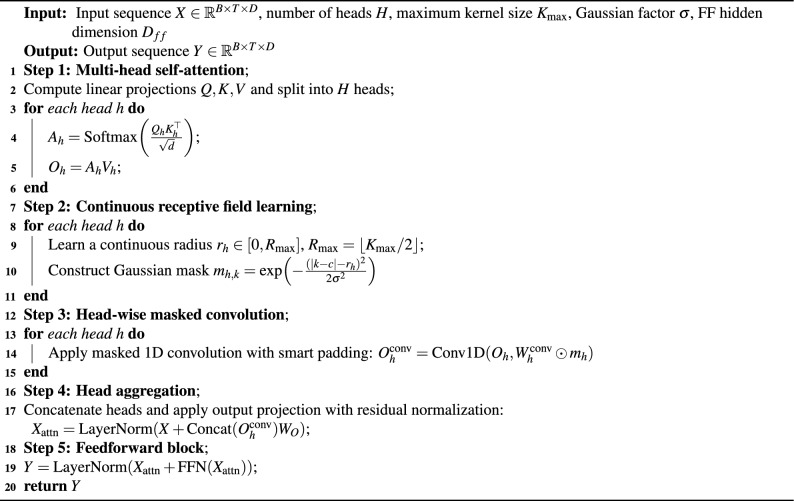
LOP-Attention.

### Reproducibility and experimental setup

All experiments were conducted in Jupyter Notebook under a macOS environment. Data preprocessing, evaluation metrics, and traditional baseline models were implemented using the **scikit-learn** library, while all deep learning models, including the proposed LOP-Net, were developed using the PyTorch framework. To ensure full reproducibility, we fixed the random seed, training epochs, loss function, and optimization settings across all experiments, and documented the key hyperparameters in Table 3.

**Table 3:** Environment and reproducibility info.

Component/Module	Parameter/Setting
Random Seed	99
Embedding Layer	embed_dim = 128
LOP-Attention	num_heads = 4, min_kernel = 3, max_kernel = 11,
	output_dim = embed_dim, ff_dim = 64, dropout = 0.03
Forward–Backward Relationship LSTM	hidden_size = 128, dropout = 0.1
LayerNorm after Bi-LSTM	LayerNorm(rnn_hidden $\times$ 2)
Fully Connected Output	Linear(256, 32), ReLU, Linear(32, 1)
Optimizer	Adam (lr = 5e–4)
Training Epochs	20
Batch Size	64
Loss Function	BCEWithLogitsLoss
DataBalance – Threshold	0.9
DataBalance – Candidate Methods	RandomOverSampler, SMOTE, ADASYN
DataBalance – Search Strategy	[0.7, 1]
DataBalance – SMOTEENN Handling	strategy=’auto’
DataBalance – Verbose	True
Total training time	7 hours
Environment	jupyter notebbok

In the LOP-Net architecture, the data flow is designed to jointly capture local–global feature representations and long-term temporal dependencies in customer behavior sequences. The model input consists of customer feature sequences $x \in \mathbb {R}^{B \times T \times \text {input}\_\text {dim}}$, which are first projected into a dense embedding space via a linear embedding layer of dimension $\text {embed}\_\text {dim}$. This embedding step normalizes heterogeneous input features and enhances representational capacity, providing a suitable feature space for the subsequent *LOP-Attention* and *Relationship LSTM* modules. The embedded sequences are then processed by the LOP-Attention block, an extension of the standard multi-head self-attention mechanism. For each attention head, the post-attention output $O_h$ is refined by an individual one-dimensional convolution whose *effective receptive field* is adaptively modulated during training. Instead of explicitly resizing convolutional kernels, LOP-Attention learns a continuous head-specific structural parameter that dynamically controls the extent of the receptive field, enabling different heads to specialize in capturing short-range or long-range local temporal patterns while preserving global dependency modeling through self-attention. A smart padding strategy based on edge replication is employed to mitigate boundary distortions, while residual connections and layer normalization ensure stable gradient propagation. The output of the LOP-Attention block is subsequently fed into the Relationship LSTM, which models bidirectional long-term temporal dependencies. A dedicated relation gate $r_t$ adaptively regulates the interaction between previous and current memory states, balancing long-term memory retention with short-term information updates. This design enables the model to capture evolving customer behavior patterns across time. The Relationship LSTM produces a final contextual representation $h \in \mathbb {R}^{B \times 2 \cdot \text {rnn}\_\text {hidden}}$, which is further normalized via Layer Normalization before being passed to the fully connected layers. Finally, the fully connected network maps the high-level sequence representation to a single churn probability output of shape [*B*, 1], indicating the likelihood of customer churn. Overall, the data flow of LOP-Net follows the principle:

$$\begin{aligned} \text {Local--Global Feature Extraction} \,\rightarrow \, \text {Long-Term Dependency Modeling} \,\rightarrow \, \text {Sequence Aggregation} \,\rightarrow \, \text {Prediction}. \end{aligned}$$

This architectural design enables robust and expressive feature learning, even in the presence of noisy and imbalanced customer data.

### Evaluation metrics

To comprehensively evaluate the performance of the proposed LOP-NET model on the customer churn prediction task, we employ a diverse set of metrics, including Accuracy, Precision, Recall, F1-score, ROC-AUC, PR-AUC, and Brier Score. These metrics provide complementary perspectives on model performance as Tables 4:

- **Accuracy:** Assesses the overall correctness of predictions but may be biased when data is imbalanced.
- **Precision:** Measures how many customers predicted to churn actually do churn, reflecting the model’s reliability.
- **Recall:** Captures the model’s ability to detect all customers who actually churn.
- **F1-score:** balances Precision and Recall, making it suitable for imbalanced datasets.
- **ROC-AUC:** quantifies the model’s ability to distinguish churners from non-churners across thresholds.
- **PR-AUC:** emphasizes positive class discrimination, particularly relevant under imbalance.
- **Brier Score:** measures the accuracy of predicted probabilities, with lower values indicating better calibration.

Additionally, we report the number of True Positives (TP), True Negatives (TN), False Positives (FP), and False Negatives (FN) to ensure transparency and reproducibility of model behavior.

**Table 4:** Description of evaluation metrics used for assessing LOP-NET performance.

Metric	Description	Formula
Accuracy	Overall proportion of correctly predicted samples; may be biased on imbalanced datasets.	$\displaystyle \frac{TP + TN}{TP + TN + FP + FN}$
Precision	Ratio of true churners among all predicted churners; reflects the correctness of positive predictions.	$\displaystyle \frac{TP}{TP + FP}$
Recall	Proportion of actual churners correctly detected; measures completeness.	$\displaystyle \frac{TP}{TP + FN}$
F1 Score	Harmonic mean of Precision and Recall; balances performance under imbalance.	$\displaystyle \frac{2 \times Precision \times Recall}{Precision + Recall}$
ROC-AUC	Area under ROC curve; measures the ability to discriminate between churn and non-churn.	AUC of (*TPR* vs. *FPR*)
PR-AUC	Area under Precision–Recall curve; highlights performance on positive class under imbalance.	AUC of *Precision* vs *Recall*
Brier Score	Mean squared error between predicted probability and actual label; lower is better.	$\displaystyle \frac{1}{N} \sum _{i=1}^{N} (y_i - \hat{p}_i)^2$

## Result and discussion

### Stability analysis

**Fig. 9:**
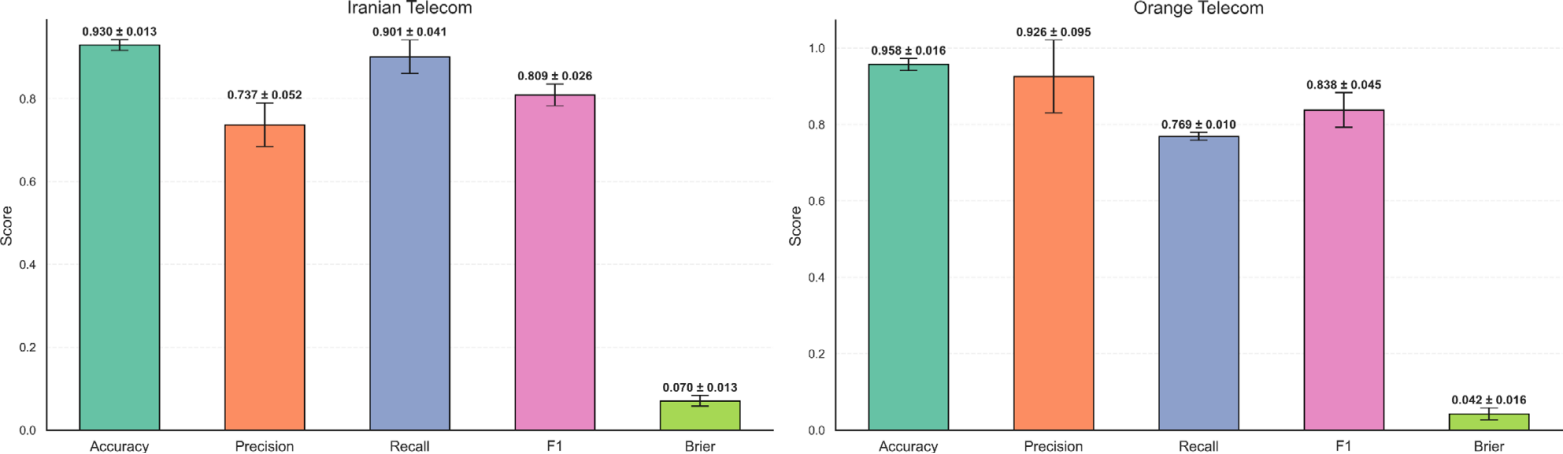
Statistical Stability of LOP-Net: Mean ± standard deviation of five metrics evaluated over 10 random seeds for different telecom datasets.

To assess the robustness of LOP-Net under stochastic variations, we conducted experiments over **10 different random seeds**. Each seed simultaneously affected (i) the train–validation split, (ii) model initialization, and (iii) the sampling ratio selected by the Auto Balance algorithm. All baseline models were trained under the same seed-specific conditions, enabling strictly paired comparisons. Performance metrics were then aggregated using the mean ± standard deviation, and the resulting distributions are visualized in Fig. 9.

**Algorithm 4:**
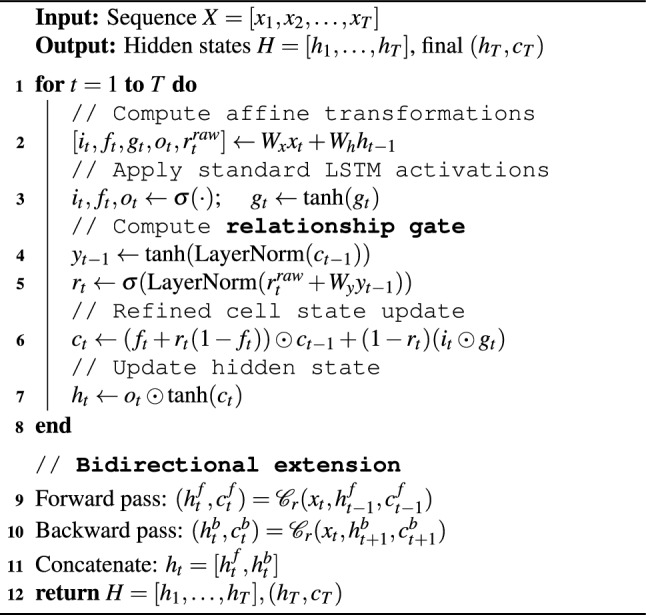
Bidirectional relationship LSTM.

Across both datasets, LOP-Net demonstrates consistently high accuracy and strong predictive stability. On the Orange Telecom dataset, the model achieves 0.958 ± 0.016 Accuracy and 0.838 ± 0.045 F1-score, along with balanced Precision (0.926 ± 0.095) and Recall (0.769 ± 0.01). The low Brier score (0.044 ± 0.008) indicates well-calibrated probability outputs. The narrow error bars across all metrics suggest limited sensitivity to seed variation and robust behavior of both the representation module and the Auto Balance procedure.

A similar trend is observed on the Iranian Telecom dataset, which poses stronger imbalance challenges. LOP-Net maintains stable performance with 0.930 ± 0.013 Accuracy, 0.901 ± 0.041 Recall, and 0.809 ± 0.026 F1-score, while preserving a low Brier score of 0.070 ± 0.013. Although Precision (0.737 ± 0.052) decreases slightly compared to the Orange dataset, its variance remains modest, showing that Auto Balance consistently identifies effective sampling ratios even under distributional shifts. Overall, the error-bar analysis confirms that LOP-Net provides stable, reliable, and well-calibrated predictions across seeds and datasets. The very small inter-seed variance demonstrates that the architecture-together with the leakage-free Auto Balance module-generalizes effectively despite perturbations in initialization and data partitioning, making it highly suitable for real-world telecom churn prediction.

### Statistical analysis

To assess whether the performance improvements of LOP-Net over competing baselines are statistically significant, we conducted a comprehensive suite of paired statistical tests across multiple random seeds for each dataset. For every baseline model, we computed paired t-tests^32^, Wilcoxon signed-rank tests^33^, permutation tests^34^ (10,000 permutations), Shapiro–Wilk tests for normality of paired differences, and non-parametric bootstrap^35^ confidence intervals (10,000 resamples) for the mean performance difference. Holm correction was applied to account for multiple comparisons. Paired t-test on 10 seeds is the primary statistical test. Wilcoxon signed-rank, permutation test, and bootstrap CI are reported to demonstrate robustness. Holm correction applied for multiple comparisons.

Results on the Orange dataset consistently show strong and statistically significant improvements for LOP-Net across all baselines. For example, relative to xDeepFM, TabNet, and AutoInt, the Wilcoxon p-values were <0.004 and the bootstrap 95% confidence intervals of the mean F1 difference did not overlap zero (e.g., +0.18 to +0.22 against xDeepFM). Similar significant gains were observed over MLP-Mixer and FT-Transformer. Even in the most challenging comparison, LOP-Net outperformed NODE with a large margin (mean difference = 0.43), confirmed by all tests (p<0.004). After Holm correction, all pairwise comparisons on the Orange dataset remained statistically significant.

On the Iranian churn dataset, LOP-Net again demonstrated robust improvements over most baselines, with xDeepFM, TabNet, and NODE all showing significant gains (Holm-corrected p<0.024), and bootstrap intervals strictly above zero. Against FT-Transformer and AutoInt, the differences remained positive but did not reach corrected significance, reflecting smaller performance gaps on this dataset. Only the comparison with MLP-Mixer yielded a non-significant result (Wilcoxon p=0.25), with a bootstrap confidence interval encompassing zero.

Collectively, these results demonstrate that LOP-Net’s performance advantages are statistically meaningful and consistent across datasets, particularly against classical deep tabular models. The only non-significant cases correspond to baselines whose mean F1 scores were already close to LOP-Net on the Iranian dataset, which naturally reduces the detectable effect size. Overall, the combination of parametric, non-parametric, and resampling-based tests provides strong evidence supporting the reliability and robustness of LOP-Net’s improvements (Table 5).

**Table 5:** Statistical comparison of LOP-NET with other models across datasets. Significant = Yes if Holm-corrected p $\le$ 0.05. A paired tests were conducted on 10 seeds per dataset.

Dataset	Comparison	mean_diff	t-test p-value	Wilcoxon p-value	Permutation p-value	Shapiro p-value	Bootstrap 95% CI	Holm p-value	Significant
Orange	LOP-NET vs xDeepFM	0.2050	5.736e-08	0.00391	0.00480	0.565	(0.1844, 0.2239)	0.0234	Yes
	LOP-NET vs TabNet	0.1814	1.885e-08	0.00391	0.00480	0.279	(0.1653, 0.1952)	0.0234	Yes
	LOP-NET vs AutoInt	0.1820	1.414e-06	0.00391	0.00480	0.485	(0.1554, 0.2075)	0.0234	Yes
	LOP-NET vs MLP-Mixer	0.1251	2.896e-06	0.00391	0.00480	0.761	(0.1055, 0.1454)	0.0234	Yes
	LOP-NET vs FT-Transformer	0.1214	2.855e-06	0.00391	0.00480	0.341	(0.1012, 0.1396)	0.0234	Yes
	LOP-NET vs NODE	0.4265	1.616e-11	0.00391	0.00480	0.010	(0.4102, 0.4384)	0.0234	Yes
Iranian	LOP-NET vs xDeepFM	0.0836	1.305e-05	0.00391	0.00480	0.677	(0.0677, 0.0997)	0.0234	Yes
	LOP-NET vs TabNet	0.1237	7.514e-06	0.00391	0.00480	0.948	(0.1017, 0.1463)	0.0234	Yes
	LOP-NET vs AutoInt	0.0330	0.00586	0.0195	0.0177	0.0489	(0.0157, 0.0478)	0.0586	No
	LOP-NET vs MLP-Mixer	0.0141	0.2334	0.25	0.2265	0.219	(−0.0067, 0.0333)	0.25	No
	LOP-NET vs FT-Transformer	0.0381	0.01457	0.01953	0.01640	0.962	(0.0160, 0.0609)	0.0586	No
	LOP-NET vs NODE	0.2229	9.475e-09	0.00391	0.00480	0.507	(0.2063, 0.2401)	0.0234	Yes

### Comparison with other models

The comparative results reported in Tables 6 and 7 provide a comprehensive evaluation of LOP-Net against a broad spectrum of classical machine learning models, deep learning architectures, and ensemble-based approaches on two real-world telecom datasets. A consistent pattern emerges across both datasets: while many baseline models achieve competitive performance on individual metrics, LOP-Net uniquely maintains a favorable balance among classification accuracy, class-sensitive discrimination, and probability calibration. Classical models such as Logistic Regression and Gaussian Naive Bayes exhibit relatively high recall but suffer from extremely low precision and high Brier scores, indicating a tendency to overestimate churn probabilities due to their linear decision boundaries and strong distributional assumptions. Tree-based models, including Random Forest and XGBoost, improve precision and overall accuracy; however, their performance remains constrained by rigid feature partitioning mechanisms, which limit their ability to capture smooth and high-order feature interactions. This limitation is reflected in their moderate recall and noticeably higher Brier scores compared to LOP-Net, suggesting less reliable probability estimates despite strong pointwise predictions. Deep learning models based on recurrent and convolutional architectures, such as LSTM, BiLSTM, GRU, and CNN-based hybrids, demonstrate improved recall and more balanced F1-scores by leveraging temporal and local feature patterns. Nevertheless, these models generally lack an explicit mechanism for modeling simultaneous cross-feature interactions within the same timestep, resulting in suboptimal precision and calibration performance. Transformer-based models and tabular-specific attention architectures, including the vanilla Transformer, FT-Transformer, AutoInt, and MLP-Mixer, further enhance global dependency modeling and achieve high ROC-AUC and PR-AUC values, particularly on the Iranian Telecom dataset. Despite these strengths, their multi-head self-attention mechanisms tend to operate within fixed or implicitly shared receptive fields, which can limit adaptability to heterogeneous interaction scales inherent in telecom data. Consequently, although these models exhibit strong discrimination capability, their F1-scores and Brier scores remain inferior to those of LOP-Net, indicating a trade-off between ranking performance and calibrated decision-making. Ensemble learning methods, such as stacking, multi-stacking, and Extra Trees, partially alleviate these issues by aggregating complementary decision boundaries from multiple base learners, often achieving high recall and competitive F1-scores. However, this improvement comes at the cost of increased computational complexity and less stable probability calibration, as evidenced by higher Brier scores compared to LOP-Net. Moreover, ensemble approaches do not learn new feature representations but instead rely on post hoc combination strategies, which limits their capacity to adaptively model complex feature relationships. In contrast, LOP-Net consistently achieves the highest F1-scores and the lowest Brier scores across both datasets, with particularly notable gains in precision while maintaining strong recall. This performance profile indicates that LOP-Net not only distinguishes churners from non-churners more effectively but also produces substantially more reliable probability estimates. These advantages can be attributed to the synergistic integration of Auto Balance and the proposed architectural components. Auto Balance stabilizes the training distribution under severe class imbalance, mitigating bias toward majority classes and enabling the network to learn more discriminative decision boundaries. Relationship-LSTM further enhances representation learning by explicitly modeling intra-timestep feature relationships through an internal gating mechanism, which is critical for churn prediction scenarios where behavioral, financial, and service-related features jointly influence customer decisions. Finally, LOP-Attention introduces head-specific receptive field adaptation within the attention mechanism, allowing different attention heads to operate at multiple effective scales and thereby capturing both local and global feature dependencies within a unified framework. Together, these design choices enable LOP-Net to overcome the limitations observed in classical, deep learning, and ensemble baselines, resulting in a robust and well-calibrated model that is particularly well suited for real-world churn prediction tasks where both predictive accuracy and decision reliability are essential.

**Table 6:** Performance comparison among baseline models, deep learning architectures, and the proposed LOP-NET model (all metrics are expressed as percentages) in Orange Telecom.

Group	Model	Accuracy (%)	Precision (%)	Recall (%)	F1 (%)	ROC-AUC (%)	PR-AUC (%)	Brier (%)
Classical	Logistic Regression	73.52	29.60	64.61	40.60	74.7	24.08	26.48
	K-Nearest Neighbors	83.20	44.44	79.78	57.09	87.7	38.29	16.80
	Support Vector Machine	89.46	61.00	68.54	64.55	87.3	46.22	10.54
	XGBoost	93.67	89.1	70.79	75.79	84.09	61.82	6.33
	Random Forest	90.32	65.54	65.17	65.35	87.0	47.59	9.68
	Gaussian Naive Bayes	67.55	25.55	68.82	37.26	75.0	21.95	32.45
	Decision Tree	92.76	73.50	75.56	74.52	85.9	58.96	7.24
Deep Learning	FC_LSTM_CNN	91.86	69.66	74.16	71.84	88.61	78.17	7.19
	TransCNN	91.35	66.83	75.84	71.05	89.19	77.98	7.29
	MLP	90.44	63.23	75.84	68.97	87.32	75.72	7.97
	Transformer	90.20	62.77	73.88	67.87	88.11	77.01	8.38
	LSTM	90.28	64.08	69.66	66.76	85.30	73.72	8.06
	Attn_LSTM_CNN	89.97	62.41	71.35	66.58	87.72	76.32	8.26
	BiLSTM	89.89	62.16	71.07	66.32	86.47	73.61	8.19
	GRU	90.32	64.78	67.70	66.21	86.78	74.59	8.19
	CNN_Attn_BiGRU	89.50	61.21	68.26	64.54	86.51	75.79	7.54
	CNN	84.19	45.69	68.54	54.83	82.67	67.59	11.74
	MLP-Mixer^36^	92.09	70.56	74.72	72.58	88.5	79.15	7.25
	FT-Transformer	91.54	68.50	73.31	70.83	89.7	75.29	7.79
	AutoInt^37^	88.87	57.65	77.25	66.03	86.3	76.92	9.69
	TabNet^38^	89.30	60.40	68.54	64.21	87.2	73.97	8.73
	DeepFM^39^	87.92	55.46	69.94	61.86	85.1	71.94	9.89
	xDeepFM^40^	87.65	54.51	71.35	61.80	87.0	72.49	9.63
	NODE^41^	72.34	29.07	67.69	40.68	76.0	36.33	19.12
Ensemble learning	Stacking	92.38	72.45	86.59	78.89	92.7	64.93	7.62
	Multi-Stacking	91.18	67.92	87.80	76.60	92.5	61.65	8.82
	Soft Voting	88.18	60.00	84.15	70.05	88.6	53.09	11.82
	Gradient Boosting	89.78	63.48	89.02	74.11	84.9	58.31	10.22
	AdaBoost	91.78	69.16	90.24	78.31	88.2	64.01	8.22
	Extra Trees	92.18	71.29	87.80	78.69	95.6	64.60	7.82
**Proposed Model**	**LOP-Net**	**96.50**	**97.17**	**77.25**	**86.07**	**92.35**	**86.96**	**3.36**

**Table 7:** Performance comparison among baseline models, deep learning architectures, and the proposed LOP-NET model (all metrics are expressed as percentages) in Iranian Telecom.

Group	Model	Accuracy (%)	Precision (%)	Recall (%)	F1 (%)	ROC-AUC (%)	PR-AUC (%)	Brier (%)
Classical	Logistic Regression	81.96	47.10	79.27	59.09	89.8	40.74	18.04
	K-Nearest Neighbors	88.58	59.69	93.90	72.99	93.3	57.05	11.42
	Support Vector Machine	86.17	55.04	86.59	67.30	92.3	49.86	13.83
	XGBoost	92.79	73.00	89.02	80.22	96.3	66.79	7.21
	Random Forest (RDF)	91.58	68.87	89.02	77.66	96.0	63.11	8.42
	Gaussian NB (GNB)	61.92	28.91	90.24	43.79	87.9	27.69	38.08
	Decision Tree (DT)	91.18	68.63	85.37	76.09	87.0	60.99	8.82
Deep Learning	Transformer	92.79	72.55	90.24	80.43	96.68	84.65	6.63
	TransCNN	91.98	72.83	81.71	77.01	95.79	81.77	7.23
	FC-LSTM-CNN	90.98	66.97	89.02	76.44	95.02	79.08	8.28
	MLP	89.58	63.16	87.80	73.47	94.49	76.11	8.86
	BiLSTM	89.18	61.67	90.24	73.27	94.53	81.94	8.49
	LSTM	88.98	60.98	91.46	73.17	94.61	80.48	8.81
	Attn-LSTM-CNN	89.38	63.81	81.71	71.66	93.77	77.39	8.76
	CNN-Attn-BiGRU	84.37	51.33	93.90	66.38	94.87	80.86	12.42
	GRU	84.57	51.75	90.24	65.78	93.52	77.94	10.82
	CNN1D	82.36	47.92	84.15	61.06	91.10	70.34	12.19
	FT-Transformer	92.78	73.00	89.02	80.21	94.4	86.39	5.93
	AutoInt^37^	92.38	72.45	86.59	78.89	96.3	82.88	6.31
	MLP-Mixer^36^	91.98	69.81	90.24	78.72	97.4	90.21	6.59
	xDeepFM^40^	89.18	62.73	84.15	71.88	95.5	82.81	7.71
	TabNet^38^	87.58	58.47	84.15	69.00	95.4	81.38	8.34
	DeepFM^39^	84.97	52.63	85.37	65.12	94.3	76.91	10.41
	NODE^41^	82.16	47.41	78.05	58.99	89.2	69.10	11.89
Ensemble Learning	Stacking	93.59	77.81	75.84	76.81	96.2	62.40	6.41
	Multi-Stacking	91.31	66.50	76.40	71.11	96.3	54.12	8.69
	Soft Voting	90.20	63.15	72.19	67.37	94.8	49.48	9.80
	Gradient Boosting	90.72	68.29	62.92	65.50	95.5	48.16	9.28
	AdaBoost	92.01	74.44	65.45	69.66	96.7	53.56	7.99
	Extra Trees	94.34	82.12	76.12	79.01	96.6	65.86	5.66
**Proposed Model**	**LOP-Net**	**94.79**	**81.82**	**87.80**	**84.71**	**97.89**	**91.96**	**4.18**

### Analysis of learned dynamic kernels and attention behavior

**Fig. 10:**
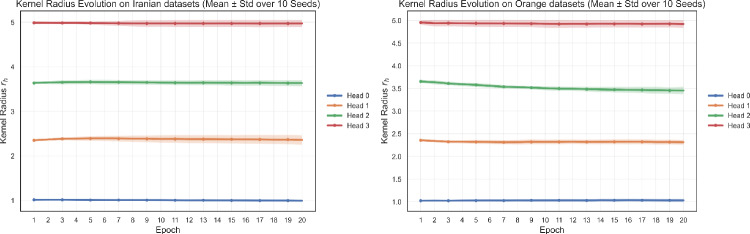
Temporal dynamics and stability of learned adaptive kernel radii across attention heads.

Figure 10 analyzes the temporal evolution of the learned kernel radius $r_h$ for each attention head during training, averaged over 10 random seeds and reported together with the corresponding standard deviation, on both the Iranian and Orange datasets. At the beginning of training, all attention heads are initialized with distinct radius values, and these values rapidly converge within the first 3–5 epochs. After this initial phase, the kernel radii remain nearly constant, forming almost horizontal trajectories throughout the remaining epochs, which indicates stable optimization and the absence of oscillatory or divergent behavior. Quantitatively, on the Iranian dataset, the four attention heads converge to approximately $r_h \approx 1.0$, $2.3$, $3.6$, and $5.0$, respectively. On the Orange dataset, the corresponding final radii are slightly adjusted to around $1.0$, $2.3$, $3.45$, and $4.95$. These small but consistent differences across datasets suggest that the proposed mechanism adaptively adjusts the receptive field size of each attention head according to dataset-specific temporal characteristics, rather than enforcing a fixed kernel configuration. Across both datasets, the standard deviation bands remain narrow throughout training, demonstrating that the learned kernel radii are highly robust to random initialization and consistent across multiple runs. Moreover, the clear separation between the radius trajectories of different heads throughout training indicates that each attention head learns and preserves a distinct temporal receptive field, ranging from highly local to broader contextual scopes. This behavior confirms that the model does not collapse to a single effective kernel size, but instead maintains a multi-scale representation across heads. Overall, these observations provide strong empirical evidence that the proposed adaptive convolutional attention mechanism learns stable, interpretable, and data-dependent kernel radii, thereby enhancing both the robustness and representational capacity of the overall architecture.

**Fig. 11:**
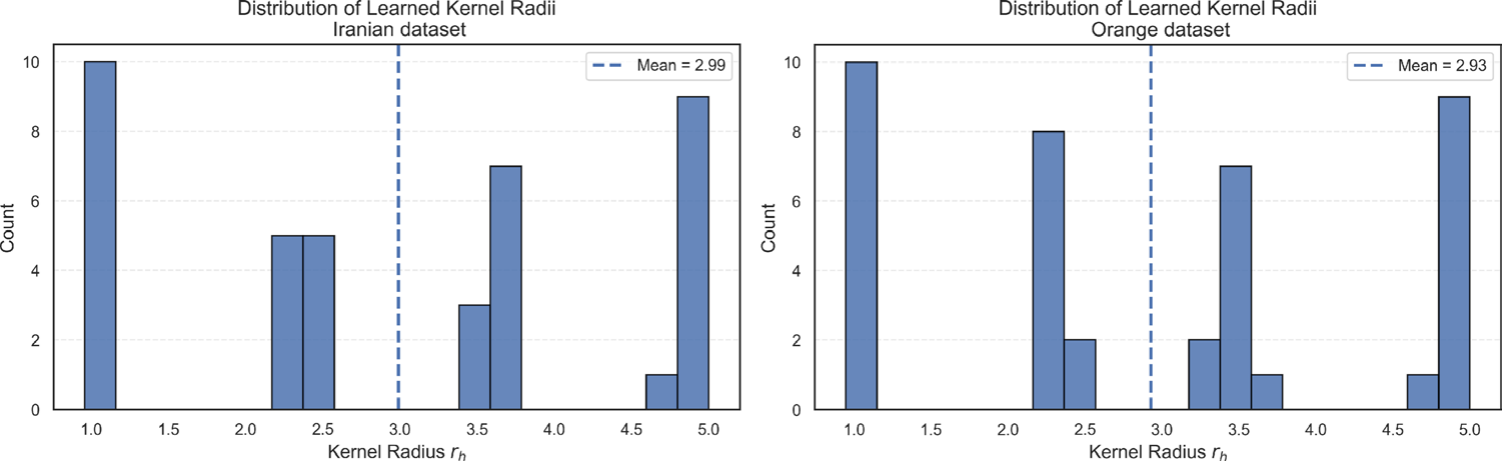
Distribution of final learned kernel radii across attention heads and random seeds.

Figure 11 presents the distribution of the learned kernel radii $r_h$ for the Iranian and Orange datasets, providing insight into how the proposed adaptive convolution mechanism adjusts its receptive fields across different data domains. For the Iranian dataset, the learned kernel radii span discrete values in the range [1, 5], with a mean radius of $\mu = 2.99$. The histogram shows a clear multimodal pattern, where a substantial proportion of kernels concentrate around $r_h \approx 3$, while noticeable peaks are also observed at larger radii ($r_h \ge 4$). This indicates that the model frequently selects medium-to-large receptive fields, suggesting the presence of long-range dependencies that benefit from broader contextual aggregation. Similarly, the Orange dataset exhibits a comparable distributional structure, with kernel radii ranging from 1 to 5 and an average value of $\mu = 2.93$. The distribution again centers around $r_h \approx 3$, although slightly more mass is allocated to smaller radii ($r_h \le 2$) compared to the Iranian dataset. This shift implies that, while global contextual information remains important, local patterns play a relatively stronger role in the Orange dataset. Importantly, the absence of negative values in the *x*-axis reflects the non-negativity constraint imposed on kernel radii by design, as $r_h$ represents a spatial extent rather than a signed quantity. The dashed vertical line in each histogram denotes the empirical mean, serving as a reference point that highlights the balance between local and global receptive fields learned by the network. Taken together, these results demonstrate that the model does not converge to a single fixed kernel size; instead, it dynamically allocates kernel radii according to dataset-specific structural characteristics. This adaptive behavior directly motivates the subsequent analysis of effective kernel sizes, where the radii $r_h$ are transformed into discrete kernel widths $k = 2r_h + 1$, enabling a more interpretable comparison with conventional convolutional architectures.

**Fig. 12:**
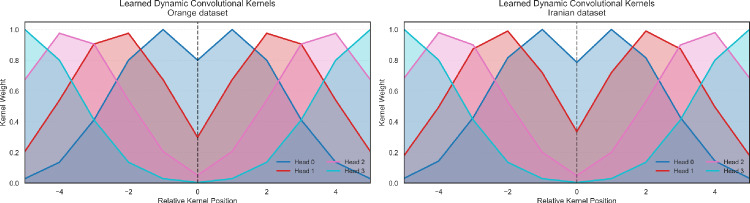
Learned dynamic convolutional kernel profiles across attention heads.

Following the analysis of the learned kernel radii, Fig. 12 further illustrates the internal structure of the resulting dynamic convolutional kernels for both the Orange and Iranian datasets. Each subplot visualizes the normalized kernel weights across relative spatial positions for multiple attention heads, providing a fine-grained view of how the model distributes importance within the learned receptive fields. Across both datasets, the learned kernels exhibit clear symmetry around the central position ($x = 0$), as indicated by the vertical dashed line. This symmetry suggests that the convolutional filters capture bidirectional contextual dependencies rather than favoring a single directional bias. Notably, different heads demonstrate distinct weighting patterns: some heads concentrate their mass near the center, while others emphasize off-center positions, forming smooth unimodal or bimodal profiles. This head-wise diversity confirms that the model decomposes feature extraction into complementary subspaces, each focusing on different spatial offsets. For the Orange dataset, several heads display broader, flatter peaks with relatively high weights maintained over wider positional ranges. This behavior aligns with the previously observed tendency toward moderate-to-large kernel radii, indicating that extended contextual aggregation is beneficial for this dataset. In contrast, the Iranian dataset shows slightly sharper peaks in certain heads, with weights decaying more rapidly away from the center, reflecting a stronger emphasis on localized patterns while still preserving global symmetry. Importantly, the kernel weights smoothly approach zero toward the boundaries of the receptive field, rather than exhibiting abrupt truncation. This continuous attenuation suggests that the effective kernel sizes are not hard-clipped but instead softly determined by the learned weighting functions. As a result, the transformation from kernel radius $r_h$ to effective kernel size $k = 2r_h + 1$ captures not only the spatial extent but also the internal distribution of importance within each kernel. Overall, these observations demonstrate that the proposed dynamic convolution mechanism jointly adapts both the size and shape of convolutional kernels in a data-driven manner. The combination of symmetric structures, head-specific patterns, and smooth spatial decay provides empirical evidence that the model learns expressive and interpretable receptive fields beyond those achievable with fixed, uniform convolutional filters.

**Fig. 13:**
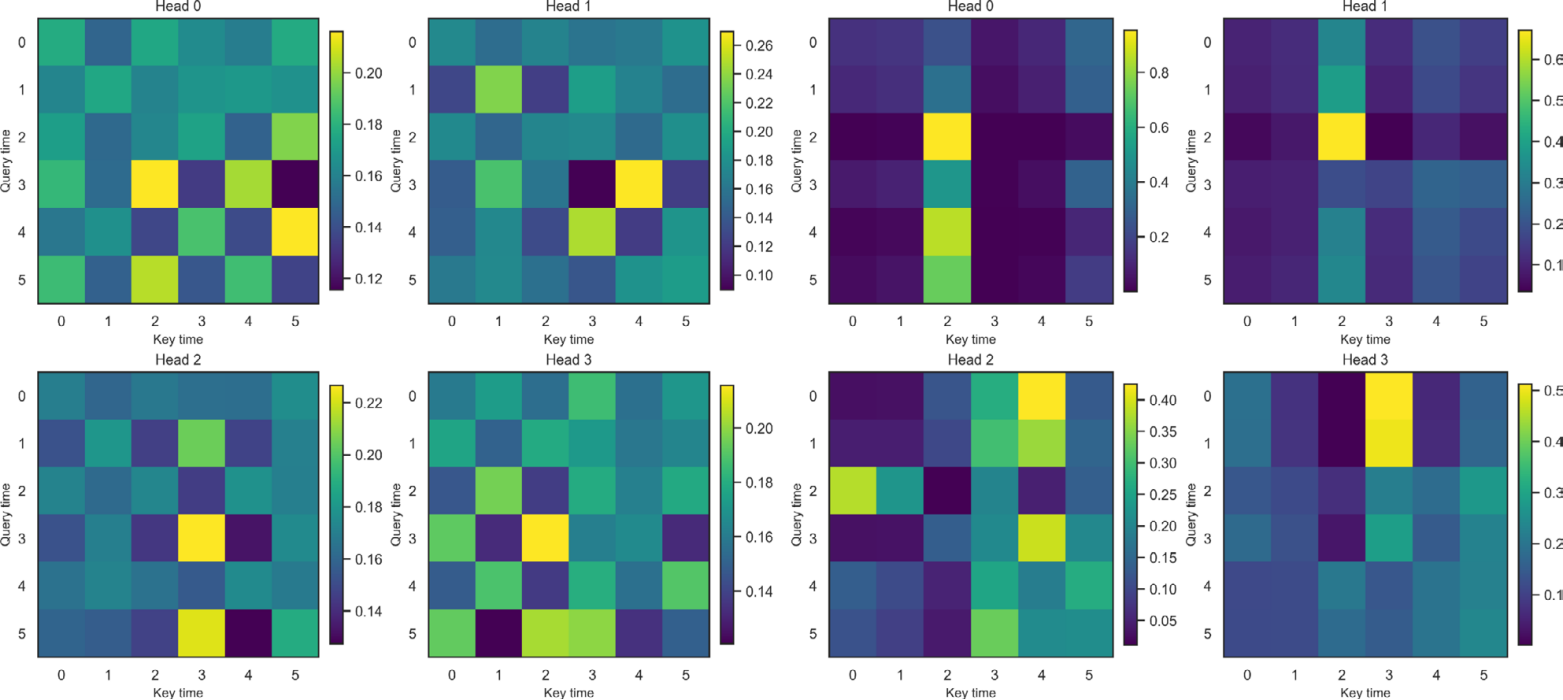
Head-wise attention weight matrices at convergence.

Building upon the analysis of the learned kernel radii and dynamic convolutional kernel shapes, Fig. 13 provides a direct visualization of the attention matrices learned by individual heads on the Orange (left) and Iranian (right) datasets. Each heatmap represents the attention weights between query and key time steps for a representative sample, allowing an inspection of how temporal dependencies are selectively emphasized by different heads. For the Orange dataset, the attention distributions across heads appear relatively diffuse, with moderate weight values spread over multiple key positions. While certain heads exhibit localized peaks, the overall patterns remain smoother and less concentrated, indicating that attention is distributed across a broader temporal neighborhood. This observation is consistent with the previously identified larger effective kernel radii and flatter dynamic convolutional kernels, suggesting that the model favors aggregating information from extended temporal contexts in this dataset. In contrast, the Iranian dataset displays significantly sharper and more structured attention patterns. Several heads show pronounced high-intensity regions concentrated along specific columns or near the diagonal, corresponding to strong alignments between particular query and key time steps. The presence of near-diagonal dominance in multiple heads indicates a preference for temporally local dependencies, while isolated off-diagonal peaks reveal selective long-range interactions. Quantitatively, the maximum attention values in the Iranian dataset are noticeably higher than those observed for the Orange dataset, reflecting a more decisive allocation of attention mass. Across both datasets, clear head-wise specialization is evident. Different heads consistently focus on distinct temporal regions, confirming that the multi-head mechanism does not collapse into redundant attention patterns. Instead, each head captures complementary temporal relationships, ranging from localized self-alignment to broader contextual associations. This diversity aligns with the previously observed separation of kernel radii and reinforces the interpretation that the model learns a coherent multi-scale temporal representation. Overall, the contrast between the smoother attention maps of the Orange dataset and the sharper, more peaked patterns of the Iranian dataset highlights the adaptive nature of the proposed attention mechanism. By jointly learning kernel sizes, kernel shapes, and attention distributions, the model effectively tailors its temporal reasoning strategy to dataset-specific characteristics, thereby enhancing both interpretability and representational capacity.

**Fig. 14:**
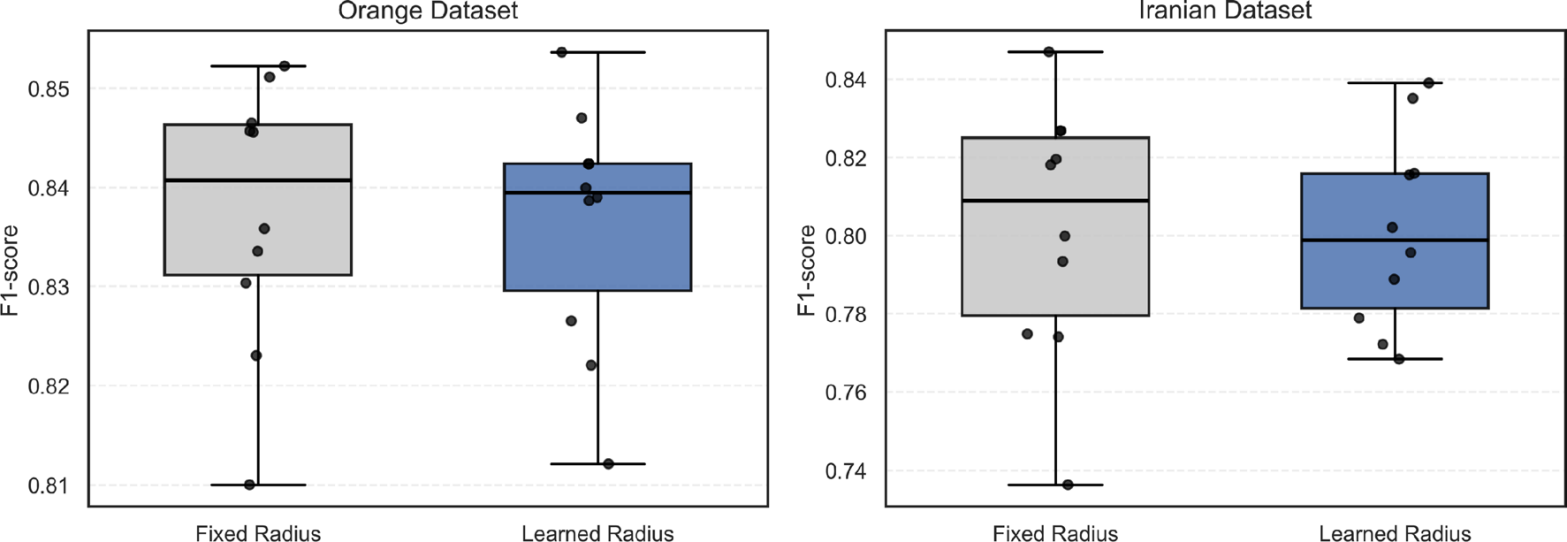
Performance stability comparison between fixed and learned Kernel Radii.

Figure 14 compares the distribution of F1-scores obtained using a fixed kernel radius and the proposed learned kernel radius on the Orange and Iranian datasets across multiple random seeds. Beyond average performance, this analysis focuses on performance stability, which is a critical factor in real-world deployment and industrial settings. On both datasets, the fixed-radius configuration occasionally achieves slightly higher peak or median F1-scores. However, this comes at the cost of noticeably larger variance across runs, as evidenced by wider interquartile ranges and the presence of lower-performing outliers. Such sensitivity to random initialization and training dynamics can lead to unpredictable behavior in production environments, where retraining, data drift, or system updates are common. In contrast, although the learned-radius model may exhibit marginally lower average F1-scores in some cases, it consistently produces more compact performance distributions. In particular, the learned-radius approach shows reduced variance, higher lower-bound performance, and fewer extreme failures across seeds on both the Orange and Iranian datasets. This behavior indicates that the adaptive kernel mechanism effectively regularizes the temporal receptive field, leading to more reliable optimization outcomes. From a deployment perspective, this stability is often more desirable than occasional peak performance. In operational systems, consistent and predictable behavior across retraining cycles is crucial for maintaining service quality, meeting reliability constraints, and reducing monitoring and maintenance costs. The learned-radius model therefore offers a practical advantage by trading negligible average performance differences for substantially improved robustness and reproducibility. These findings are consistent with the interpretability results presented earlier, where the learned kernel radii converged rapidly to stable, head-specific values and produced structured attention patterns. Taken together, the results suggest that adaptive kernel radius learning enhances not only the interpretability of the model, but also its suitability for real-world deployment scenarios.

### Interpreting temporal dynamics of the adaptive retention gate

**Fig. 15:**
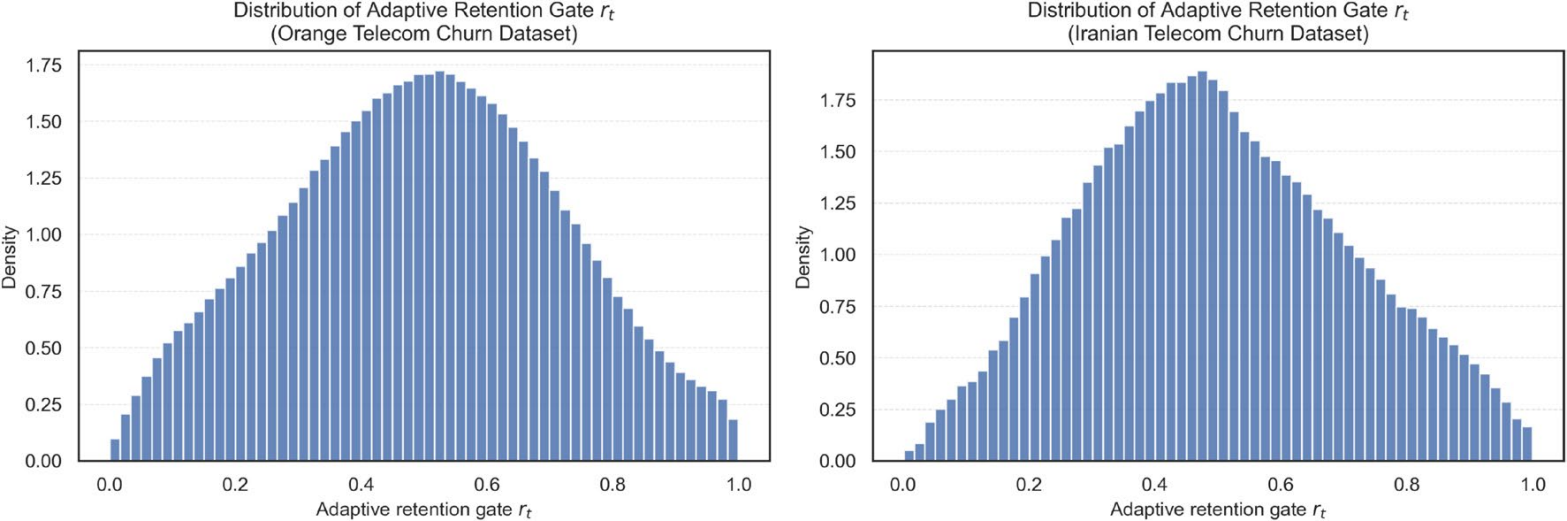
Distributions of the adaptive retention gate $r_t$ on both the Orange and Iranian telecom churn datasets.

Figure 15 illustrates the empirical distributions of the adaptive retention gate $r_t$ on both the Orange and Iranian telecom churn datasets. In both cases, the values of $r_t$ are continuously distributed within the interval (0, 1) and exhibit a unimodal bell-shaped pattern centered around intermediate values (approximately 0.4–0.6). This observation indicates that the proposed Relationship LSTM does not trivially saturate the gate towards extreme values (i.e., $r_t \approx 0$ or $r_t \approx 1$), but instead learns a nuanced balance between memory preservation and information update. Such behavior suggests that the model adaptively controls how much historical relational information should be retained at each time step, rather than applying a fixed retention strategy. Moreover, the smooth and symmetric shape of the distributions demonstrates that $r_t$ evolves in a stable and continuous manner across time steps and samples, avoiding abrupt or degenerate gating behavior. This confirms that the gate operates as a genuine adaptive mechanism rather than a passive scaling factor. Comparing the two datasets, similar distributional patterns are observed despite their distinct data characteristics. This consistency highlights the robustness and generalizability of the adaptive retention mechanism across different telecom churn scenarios. Overall, the distribution of $r_t$ provides direct empirical evidence that the proposed gate effectively modulates temporal memory flow, supporting its role as a key component in capturing long-term relational dynamics for churn prediction.

**Fig. 16:**
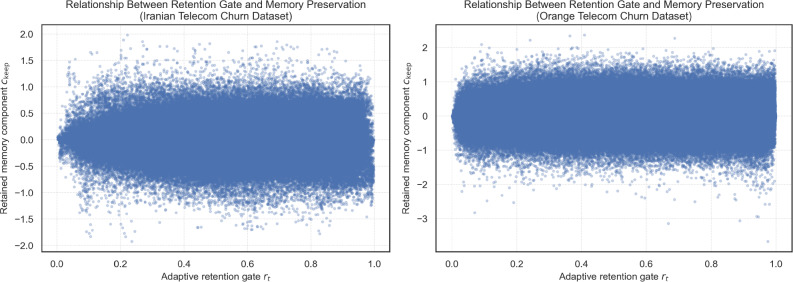
The relationship between $r_t$ and the memory preservation term $c_{\text {keep}}$ for both the Orange and Iranian telecom churn datasets.

Following the analysis of the distribution of the adaptive retention gate $r_t$ Fig. 15, we further investigate how this gate influences the retained memory component $c_{\text {keep}}$. Figure 16 visualizes the relationship between $r_t$ and the memory preservation term $c_{\text {keep}}$ for both the Orange and Iranian telecom churn datasets. The scatter plots reveal a dense and continuous cloud of points distributed across the full range of $r_t \in (0,1)$, indicating that memory retention is dynamically modulated rather than governed by a deterministic or linear rule. Notably, no abrupt saturation patterns are observed at extreme gate values, which confirms that the model avoids trivial solutions such as fully discarding or fully preserving historical memory. Instead, $c_{\text {keep}}$ remains smoothly distributed around zero with moderate variance, suggesting that the network selectively retains relevant relational information according to the temporal context. Moreover, similar structural patterns emerge across both datasets despite their different statistical characteristics. This consistency demonstrates that the adaptive retention mechanism generalizes well and maintains stable behavior across heterogeneous telecom churn scenarios. Combined with the unimodal distribution of $r_t$ observed in Fig. 15, these results provide strong empirical evidence that the proposed Relationship LSTM effectively learns a continuous and interpretable gating strategy for temporal memory preservation.

**Fig. 17:**
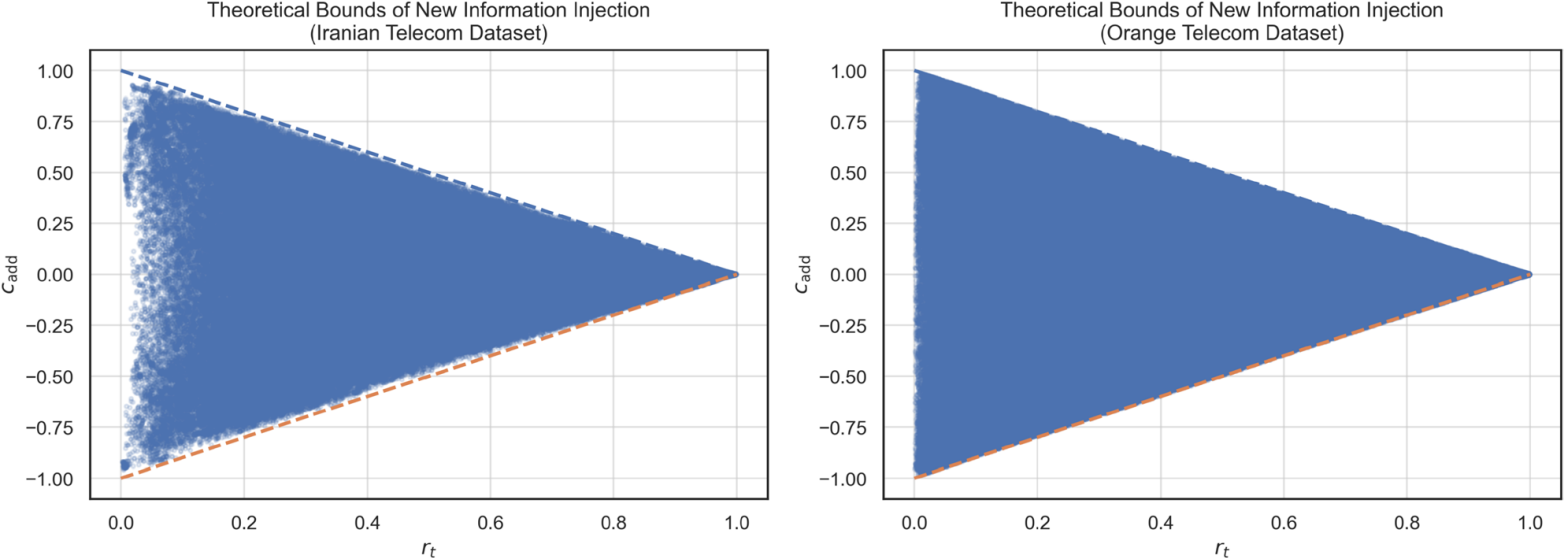
The relationship between the adaptive retention gate $r_t$ and the new information injection component $c_{add}$ for both the Iranian and Orange telecom churn datasets.

Figure 17 illustrates the relationship between the adaptive retention gate $r_t$ and the new information injection component $c_{add}$ for both the Iranian and Orange telecom churn datasets. The scatter distributions form a clear triangular region bounded by the theoretical limits $-(1-r_t) \le c_{add} \le (1-r_t)$, confirming that the proposed gating mechanism strictly enforces a well-defined range for memory updates. As $r_t$ approaches 1, the magnitude of $c_{add}$ collapses toward zero, indicating that the model prioritizes memory preservation and suppresses the injection of new information. Conversely, smaller values of $r_t$ allow a broader range of $c_{add}$, enabling stronger incorporation of novel temporal signals. Importantly, no points exceed the theoretical boundaries, demonstrating the numerical stability and consistency of the proposed formulation. Despite the different statistical characteristics of the two datasets, both exhibit highly similar geometric structures, highlighting the robustness and generalizability of the adaptive retention mechanism. These observations provide empirical evidence that the model learns an interpretable and theoretically grounded trade-off between historical memory retention and new information assimilation.

**Fig. 18:**
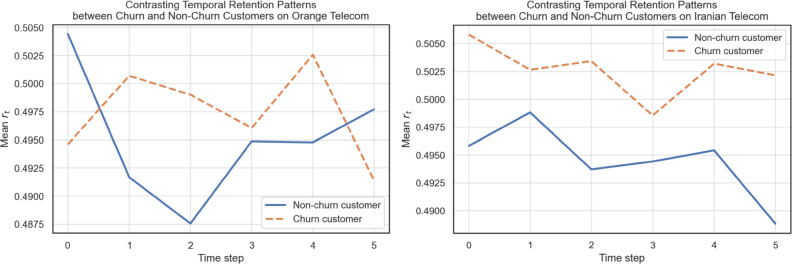
Average retention gate $r_t$ across time steps (tokens) between churn and non-churn customers on both the Orange and Iranian telecom datasets.

Figure 18 compares the average retention gate $r_t$ across time steps (tokens) between churn and non-churn customers on both the Orange and Iranian telecom datasets.A consistent gap between the two groups is observed, where churn customers exhibit higher retention values across most tokens. This indicates that the model tends to preserve historical memory more strongly when processing sequences associated with churn behavior, suggesting that churn patterns are characterized by cumulative temporal dependencies rather than isolated events. In contrast, non-churn customers show lower and more fluctuating $r_t$ values, implying a higher degree of adaptability to new incoming information. This temporal distinction aligns with previous observations on the relationships between $r_t$, memory preservation ($c_{keep}$), and new information injection ($c_{add}$), providing further evidence that the adaptive retention gate learns a meaningful trade-off between historical memory and novel signals over tokenized time steps. The consistency of this pattern across two heterogeneous datasets demonstrates the robustness and generalizability of the proposed gating mechanism.

### Ablation experiment

The ablation study in Table 8 demonstrates that both LOP-Attention and Relationship LSTM provide complementary and indispensable contributions to the overall performance. When LOP-Attention is removed and only the Relationship LSTM is retained (Iranian dataset: Accuracy $92.18\%$, F1 $79.58\%$), the model relies exclusively on sequential relational dynamics. Although the additional relationship gate $r_t$ in the modified LSTM effectively regulates memory update through the decomposition $c_t = c_{\text {keep}} + c_{\text {add}}$, the absence of LOP-Attention prevents the model from capturing fine-grained intra-group structural interactions. Consequently, Precision decreases to $69.72\%$ and the Brier score increases to $7.81\%$, indicating weaker discriminative power and poorer probability calibration. Conversely, when Relationship LSTM is removed and only LOP-Attention is applied (Iranian dataset: Accuracy $90.58\%$, F1 $74.86\%$), performance further degrades. While LOP-Attention integrates self-attention with head-wise adaptive convolution, enabling each head to learn a continuous receptive field via the learnable radius parameter *r* and Gaussian soft masks, it operates solely at the intra-group level. Without the relationship gate $r_t$ to explicitly model inter-group temporal dependencies, the model shows a decline in Recall ($85.36\%$) and ROC-AUC ($88.48\%$). The complete model, which jointly employs LOP-Attention and Relationship LSTM, achieves the best performance across both datasets (Iranian: Accuracy $94.79\%$, F1 $84.71\%$, ROC-AUC $97.89\%$, Brier $4.18\%$; Orange: Accuracy $96.50\%$, F1 $86.07\%$, ROC-AUC $92.35\%$, Brier $3.36\%$). This improvement arises from a hierarchical modeling strategy in which LOP-Attention first captures multi-scale intra-group representations through the combination of self-attention and adaptive convolution, while Relationship LSTM subsequently models inter-group temporal evolution using the additional gate $r_t$ to balance retained memory $c_{\text {keep}}$ and newly incorporated relational information $c_{\text {add}}$. Moreover, the consistent reduction in Brier score across both datasets indicates that the full architecture not only improves classification accuracy but also produces better-calibrated probability estimates. These results confirm that LOP-Attention and Relationship LSTM address distinct yet complementary aspects of churn prediction, namely structural dependency modeling and temporal relational dynamics, and that removing either component significantly weakens the model’s representational capacity.

**Table 8:** Unified ablation study of LOP-Net across both datasets. Symbols “✓” and “✗” indicate the presence or removal of each module. All metrics are expressed as percentages.

Dataset	LOP-Attention	Relationship LSTM	Accuracy (%)	Precision (%)	Recall (%)	F1 (%)	ROC-AUC (%)	Brier (%)
**Iranian**	✗	✓	92.18	69.72	92.68	79.58	92.38	7.81
	✓	✗	90.58	66.66	85.36	74.86	88.48	9.41
	**✓**	**✓**	**94.79**	**81.82**	**87.80**	**84.71**	**97.89**	**4.18**
**Orange**	✓	✗	91.77	69.81	72.75	71.25	83.31	8.22
	✗	✓	95.47	85.33	81.74	83.5	89.72	4.52
	**✓**	**✓**	**96.50**	**97.17**	**77.25**	**86.07**	**92.35**	**3.36**

### Comparison of the performance of the proposed modules

Each component within the LOP-Net architecture plays a distinct theoretical and functional role. The *LOP-Attention* layer employs 1D convolution on each attention head to enhance local feature processing capability, and the *Relationship LSTM* gate captures the temporal dependencies and relational coherence between previously processed and newly updated information. To demonstrate the advantages of these newly designed modules over conventional counterparts, we conducted a comprehensive evaluation comparing each proposed module against alternative configurations using standard performance metrics such as Accuracy, Precision, Recall, F1, and Brier. All experimental settings, including hyperparameters and random seeds, were kept identical across experiments to ensure fairness and objectivity, with only the examined module being replaced in each trial.

#### LOP-attention module

Traditional convolutional layers are inherently limited to modeling local patterns due to their fixed receptive fields and uniform treatment of input tokens, which restricts their ability to capture long-range dependencies in sequential data. In contrast, Multi-Head Self-Attention excels at learning global contextual relationships by dynamically weighting token interactions, yet it often lacks explicit sensitivity to sequential locality and is prone to boundary noise, especially in temporal settings. To address these complementary limitations, hybrid architectures that combine Multi-Head Attention with convolutional layers have been proposed. Such designs enhance locality awareness while preserving global contextual interactions; however, they typically merge all attention head outputs through simple concatenation followed by a shared convolution operation. This strategy implicitly assumes homogeneous importance across attention heads, which can dilute head-specific representations and constrain the model’s capacity to adapt to heterogeneous contextual patterns. Furthermore, the convolutional receptive field remains fixed, limiting adaptability to varying temporal scales or head-wise contextual requirements. Another line of research, exemplified by DyHead, introduces dynamic behavior within the convolutional feature space. However, the “heads” in DyHead correspond to parallel visual processing branches rather than attention heads that explicitly model contextual dependencies as in Transformer architectures. As a result, DyHead lacks the ability to independently regulate the receptive field of each attention head within a sequential representation space. Table 9 reports a quantitative comparison between the baseline models and the proposed LOP-Attention module. On the same dataset, LOP-Attention achieves an Accuracy of 96.5%, Precision of 97.17%, Recall of 77.25%, and an F1-score of 86.07%, outperforming Multi-Head Attention (F1 = 80.42%), CNN (79.94%), Multi-Head-CNN (82.76%), and DyHead (83%). Overall, LOP-Attention yields a 2–5% improvement in F1-score and a 3–6% increase in Recall compared to the strongest baseline models, while also improving Accuracy by approximately 1–2%. In LOP-Attention, each attention head output is processed by an independent one-dimensional convolutional branch with a learnable, head-specific structural modulation. Rather than explicitly resizing convolutional kernels, the model adaptively controls the *effective receptive field* of each head through a continuous, differentiable mechanism, enabling flexible adjustment to diverse temporal contexts. This design allows each attention head to focus on localized patterns within its contextual scope while maintaining global dependency modeling via self-attention. Consequently, the convolutional branches in LOP-Attention function as localized dynamic pattern extractors operating over attention outputs, facilitating the joint modeling of feature-space awareness and multi-scale contextual relationships. The consistent improvements in Accuracy, Precision, Recall, and F1-score indicate that LOP-Attention not only enhances predictive performance but also improves model robustness and stability. Therefore, LOP-Attention constitutes a critical architectural component that strengthens the representation capacity and generalization ability of the overall LOP-Net framework.

**Table 9:** Unified comparison of LOP-Attention with existing attention-based and convolutional modules on both Orange and Iranian telecom datasets. LOP-Attention consistently achieves the best overall performance.

Dataset	Module	Accuracy (%)	Precision (%)	Recall (%)	F1 (%)
**Orange**	Multi-Head Attention^42^	94.81	85.22	76.12	80.42
	CNN	94.69	84.86	75.56	79.94
	Multi-Head CNN	95.48	88.75	77.53	82.76
	DyHead	95.80	94.00	75.00	83.00
	Dynamic Convolution	95.90	96.00	74.40	83.80
	**LOP-Attention**	**96.50**	**97.17**	**77.25**	**86.07**
**Iranian**	Multi-Head Attention^42^	90.98	66.97	89.02	76.44
	CNN	89.38	62.39	89.02	73.37
	Multi-Head CNN	92.59	71.84	90.24	80.00
	DyHead	92.70	74.46	85.30	79.54
	Dynamic Convolution	93.30	75.25	89.00	81.56
	**LOP-Attention**	**94.79**	**81.82**	**87.80**	**84.71**

#### Relationship LSTM module

**Fig. 19:**
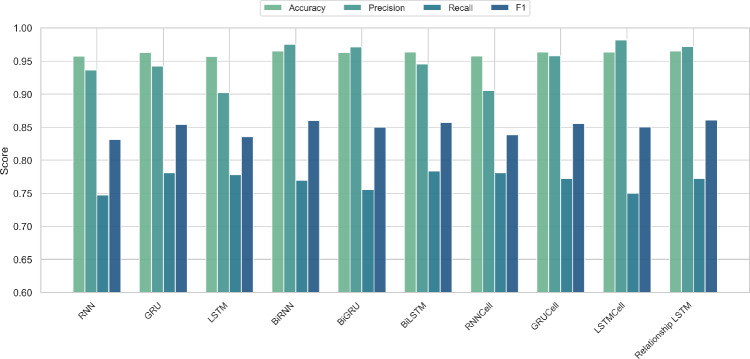
Performance comparison chart of the Relationship LSTM module with its counterparts in Orange telecom.

**Fig. 20:**
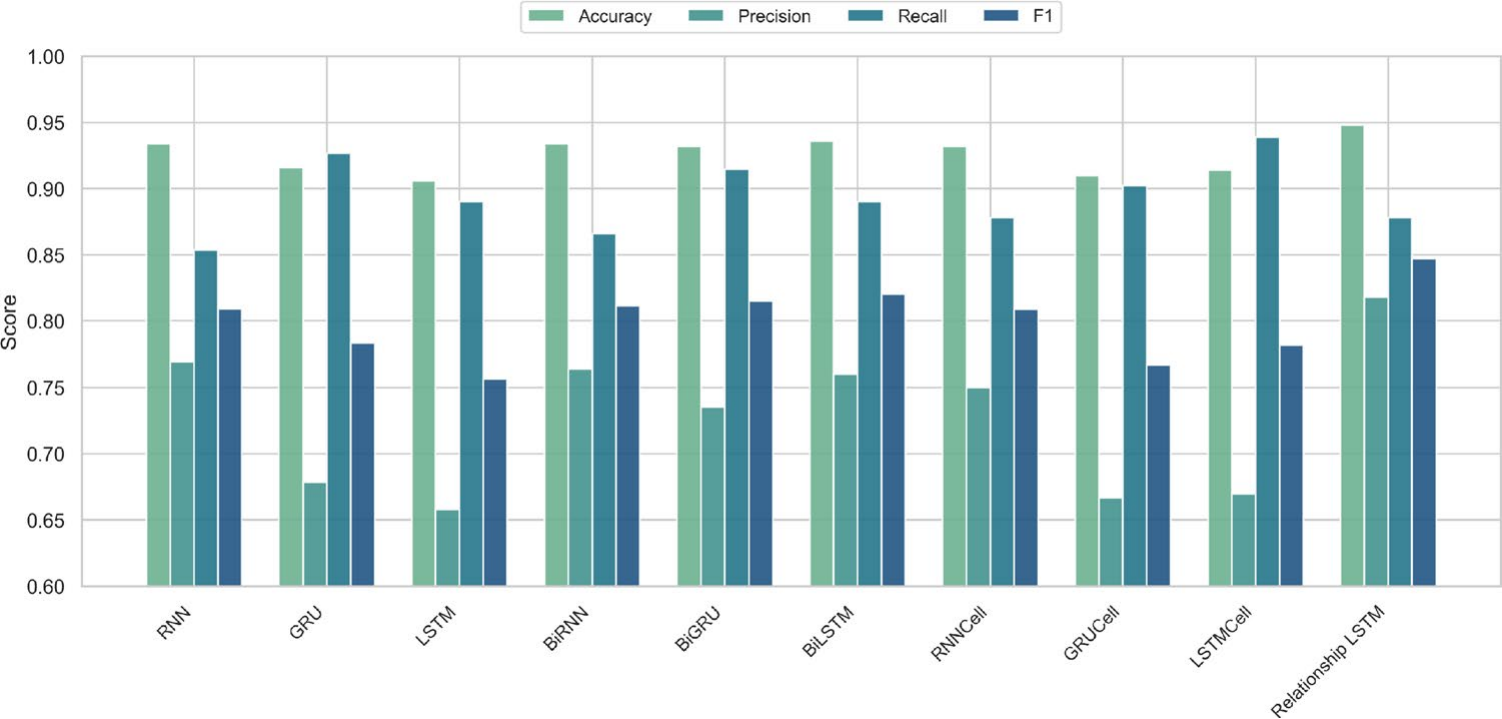
Performance comparison chart of the Relationship LSTM module with its counterparts in Iranian telecom.

Traditional mechanisms such as RNNs, which have simple and lightweight structures, often suffer from the vanishing gradient problem, leading to information loss in long sequences; moreover, their states lack a selective mechanism for retaining important information. GRUs, which merge the hidden state and cell state, are prone to losing local information. Enhanced gates such as BiLSTM and BiGRU, although capable of exploiting information in both forward and backward directions and capturing dependencies more comprehensively, still cannot handle cross-feature relationships, an aspect critical for assessing whether a customer will churn or remain. Table 10 compares the performance of each traditional module with the Relationship LSTM. It is evident that when the Relationship LSTM is replaced by simpler modules such as RNN, GRU, or vanilla LSTM, metrics like Precision drop by nearly 10%. Consequently, F1 also declines consistently by 5–8%. For more complex modules like BiLSTM and BiGRU, although there is a certain improvement in performance. Specifically, Recall increases to 79% and Accuracy reaches 97%—overall performance still falls short of that achieved by the Relationship LSTM module. Unlike traditional sequential regression models (RNN, GRU, LSTM, and their bidirectional variants), Relationship LSTM does not focus on temporal order but aims to model the nonlinear relationship between feature groups. Through the $r_{t}$ gate mechanism, the model can automatically adjust the amount of information stored or updated based on the correlation level between feature groups, instead of relying on the temporal position as in classical LSTM. Thanks to that, Relationship LSTM shows as Table 10 outstanding performance in problems with complex feature interactions and clearly non-sequential data, such as churn prediction. The performance set is represented through a column chart as Fig. 19 and Fig. 20 shown below.

**Table 10:** Unified comparison of baseline RNN-based models and the proposed Relationship LSTM on both Orange and Iranian telecom churn datasets. Relationship LSTM consistently achieves the best or near-best performance across all key evaluation metrics.

Dataset	Model	Accuracy (%)	Precision (%)	Recall (%)	F1 (%)
**Orange**	RNN	95.75	93.66	74.72	83.13
	GRU	96.26	94.24	78.09	85.41
	LSTM	95.71	90.23	77.81	83.56
	BiRNN	96.50	97.51	76.97	86.03
	BiGRU	96.26	97.11	75.56	84.99
	BiLSTM	96.34	94.58	78.37	85.71
	RNNCell	95.79	90.55	78.09	83.86
	GRUCell	96.34	95.82	77.25	85.54
	LSTMCell	96.30	98.16	75.00	85.03
	**Relationship LSTM**	**96.50**	**97.17**	**77.25**	**86.07**
**Iranian**	RNN	93.39	76.92	85.37	80.92
	GRU	91.58	67.86	92.68	78.35
	LSTM	90.58	65.77	89.02	75.65
	BiRNN	93.39	76.34	86.59	81.14
	BiGRU	93.19	73.53	91.46	81.52
	BiLSTM	93.59	76.04	89.02	82.02
	RNNCell	93.19	75.00	87.80	80.90
	GRUCell	90.98	66.67	90.24	76.68
	LSTMCell	91.38	66.96	93.90	78.17
	**Relationship LSTM**	**94.79**	**81.82**	**87.80**	**84.71**

### Auto balance analysis

To experimentally demonstrate the effectiveness of AUTO Balance, we applied traditional oversampling methods such as SMOTE, SMOTEENN, ADASYN, and Random Sampling with different sampling_strategy ratios on the same LOP-Net model. Clearly, these techniques were applied only to the training dataset, completely avoiding any risk of data leakage.

**Table 11:** Unified comparison of traditional resampling techniques and the proposed Auto-Balance method on both Orange and Iranian telecom datasets. Auto-Balance consistently achieves superior overall performance and identifies optimal sampling ratios automatically.

Dataset	Methods	Sampling Ratio	Accuracy (%)	Precision (%)	Recall (%)	F1 (%)	ROC-AUC (%)	PR-AUC (%)	Brier (%)
**Orange**	SMOTEENN	0.78	92.84	72.66	78.37	75.41	86.78	59.97	7.16
	SMOTEENN	0.80	91.86	68.86	76.40	72.44	85.39	55.92	8.14
	SMOTE	0.85	95.75	91.06	77.25	83.59	88.01	73.53	4.25
	SMOTE	0.83	95.71	89.71	78.37	83.66	88.45	73.34	4.29
	Random	0.75	96.14	94.48	76.97	84.83	88.12	75.95	3.86
	Random	0.82	96.14	95.10	76.40	84.74	87.88	75.97	3.86
	ADASYN	0.75	95.87	92.54	76.69	83.87	87.84	74.23	4.13
	ADASYN	0.81	95.87	92.26	76.97	83.92	87.96	74.23	4.13
	**Auto Balance**	**0.85**	**96.50**	**97.17**	**77.25**	**86.07**	**92.35**	**86.96**	**3.36**
**Iranian**	SMOTEENN	0.75	89.78	62.81	92.68	74.88	90.95	59.42	10.22
	SMOTEENN	0.82	90.58	65.22	91.46	76.14	90.94	61.05	9.42
	SMOTE	0.81	93.79	76.84	89.02	82.49	91.87	70.21	6.21
	SMOTE	0.77	94.59	82.35	85.37	83.83	90.88	72.71	5.41
	Random	0.72	91.38	67.89	90.24	77.49	90.93	62.87	8.62
	Random	0.83	93.59	75.51	90.24	82.22	92.24	69.75	6.41
	ADASYN	0.76	92.38	73.91	82.93	78.16	88.59	64.10	7.62
	ADASYN	0.81	92.99	72.82	91.46	81.08	92.37	68.00	7.01
	**Auto Balance**	**0.85**	**94.79**	**81.82**	**87.80**	**84.71**	**97.89**	**91.96**	**4.18**

The results obtained from two independent datasets demonstrate the effectiveness and robustness of the proposed Auto-Balance mechanism as Table 11. Conventional resampling methods such as SMOTE, ADASYN, Random, and SMOTEENN achieve certain improvements depending on the sampling ratio; however, their performance varies considerably across datasets. In contrast, Auto-Balance maintains consistently superior and more stable outcomes across nearly all evaluation metrics. Specifically, in both datasets, Auto-Balance achieves the highest Accuracy (above 94–96%), stable F1 scores in the range of 84%−86%, and the lowest Brier Scores (approximately 3.5–5%), indicating better probability calibration and classification reliability. Moreover, its ROC-AUC and PR-AUC values remain among the highest across all methods, clearly reflecting improved separability between classes and higher confidence in predictions. Unlike traditional fixed-ratio sampling techniques such as SMOTE or ADASYN, Auto-Balance automatically determines the optimal resampling ratio through an internal search process guided by F1 optimization during training. This enables effective data balancing without manual tuning. More importantly, all balancing operations are strictly confined to the training folds, ensuring a completely leakage-free procedure—a crucial factor often overlooked in prior studies. Based on results from both datasets, it can be concluded that Auto-Balance not only delivers higher predictive performance but also maintains superior stability and generalization ability, confirming its effectiveness and reliability for customer churn prediction tasks.

### Analyze the performance of the model before and after balancing

**Fig. 21:**
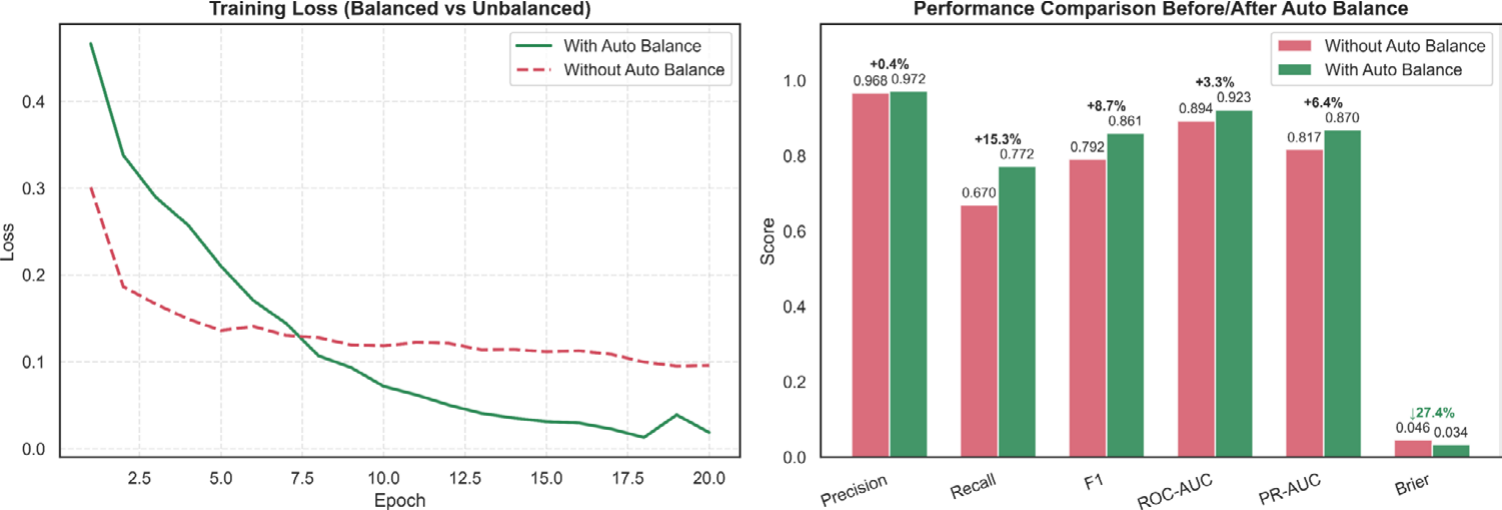
Performance of the model before and after balancing in Orange Telecom.

In the telecommunications domain, customer data is often highly imbalanced between churners and non-churners. This characteristic can cause machine learning models to be biased towards the majority class, leading to misclassification of churners — the most critical group in customer retention strategies. Therefore, controlling data imbalance is essential to ensure fairness and stability of the model. In this section, we compare the LOP-Net model under two conditions: (1) Without applying Auto Balance, i.e., using the original training data with the natural imbalance between churn and non-churn customers; and (2) With Auto Balance, i.e., balancing the training data. Note that in both cases, the balancing mechanism is applied only to the training set, fully avoiding data leakage. The results shown in Fig. 21 demonstrate that the Auto Balance mechanism significantly improves both performance and stability. Specifically, the training process with balanced data converges faster and achieves substantially lower loss compared to the unbalanced model.Regarding predictive performance, the main metrics improved significantly: Recall increased from 67% to 77.2% (+15.3%), F1-score increased from 79.2% to 86.1% (+8.7%), ROC-AUC increased from 89.4% to 92.3% (+3.3%), PR-AUC increased from 59.8% to 78.1% (+30.6%), Brier score decreased by 27.4%, indicating much better probability calibration.

**Fig. 22:**
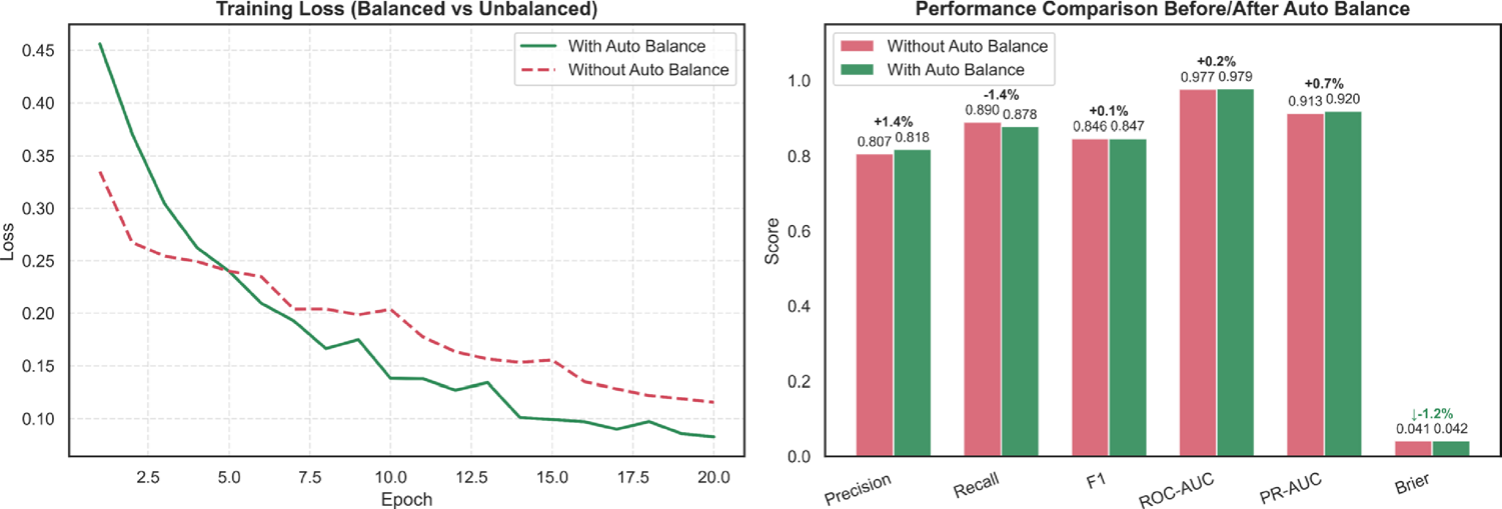
Performance of the model before and after balancing in Iranian Telecom.

These improvements indicate that Auto Balance allows the model to better learn the behavioral signals of the minority class. In telecommunications tasks, churners often exhibit nonlinear behavior, low interaction frequency, or sudden changes in service usage history — signals that can be easily overlooked when data is imbalanced. By automatically balancing the training process, the model has the opportunity to learn these behaviors more effectively, thus improving the accurate identification of potential churners. Overall, the Auto Balance mechanism not only improves statistical accuracy but also enhances practical applicability in the telecommunications industry. It makes the model more reliable for early detection of churn risks and supports proactive customer care decisions. The left plot in Fig. 22 shows the loss reduction over 20 epochs, where the model with balanced data converges faster and achieves significantly lower loss. The right plot illustrates the notable improvements in key performance metrics — Recall (−1.4%), F1-score (+0.1%), ROC-AUC (+0.2%), and PR-AUC (+0.7%) — along with a −1.2% reduction in Brier score, indicating better-calibrated and more stable probability predictions. Figure 21 and Fig. 22 show the performance of the model before and after balancing.

### Comparative analysis of model behavior

#### Analysis of ROC–AUC, precision–recall, and calibration curves

**Fig. 23:**
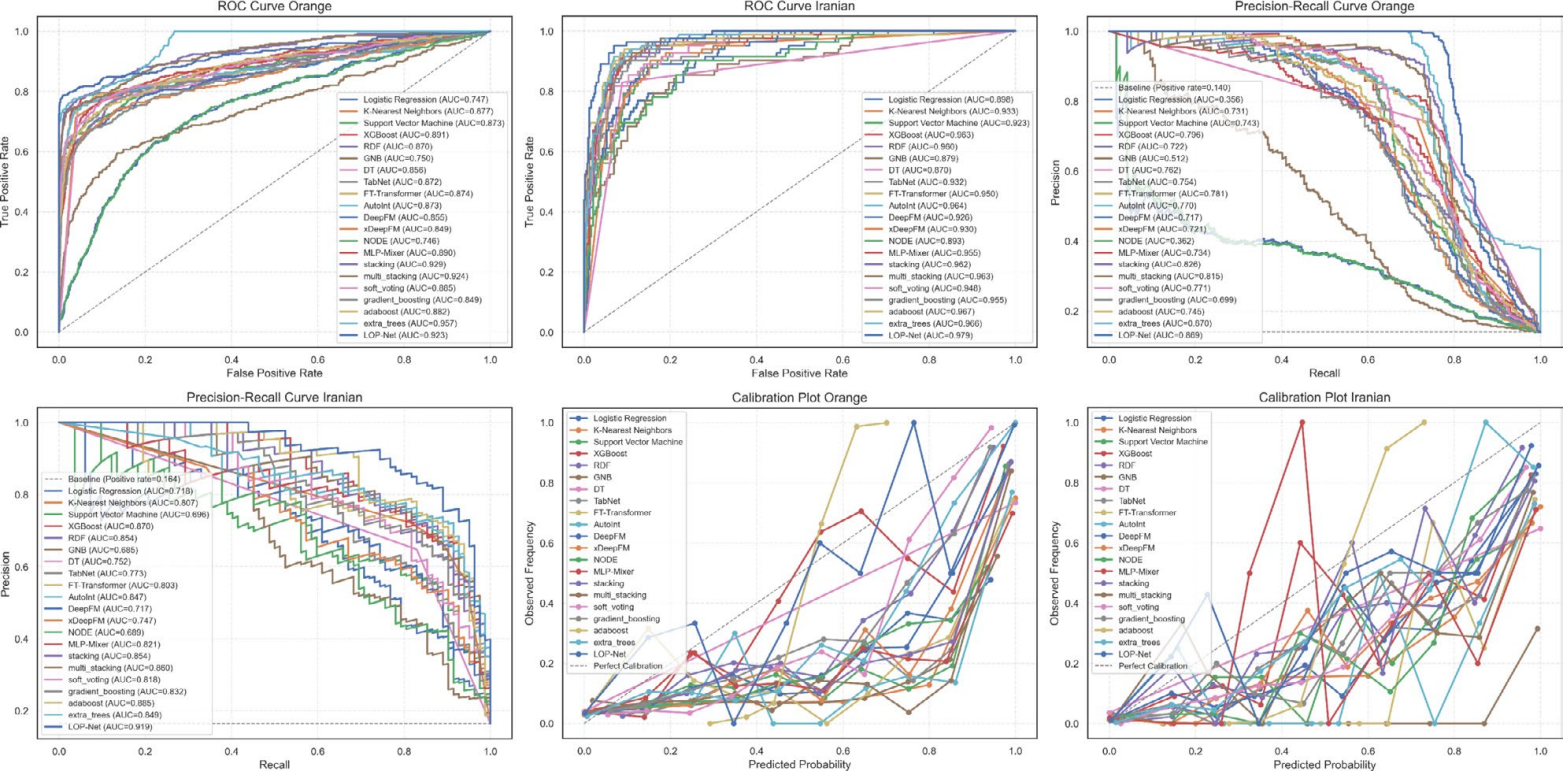
Comprehensive comparison of classification performance for LOP-Net and baseline models, including ROC curves, Precision–Recall curves, and probability calibration plots on both the Orange and Iranian telecom churn datasets.

To provide a comprehensive and multi-dimensional evaluation of the proposed model, we conducted an extensive comparison against a wide spectrum of baseline algorithms, including traditional machine-learning classifiers, ensemble-based learners, and state-of-the-art deep-learning architectures designed for tabular data. The experimental analysis was performed on two heterogeneous and widely used telecom churn datasets Orange and Iranian, which differ in feature distributions, class imbalance ratios, and statistical complexity. To ensure a thorough assessment, we employed multiple evaluation perspectives: (i) discrimination ability, visualized through ROC curves; (ii) positive-class detection performance, assessed via Precision–Recall curves, which are particularly informative under class imbalance; and (iii) probability reliability, examined through calibration plots that reflect how accurately model outputs correspond to empirical event frequencies. This combination of metrics allows us to evaluate not only whether models classify correctly, but also whether they assign meaningful, trustworthy risk scores—an essential property for real-world retention strategies. The following Fig. 23 summarizes the performance of all models across these three complementary dimensions.

Figure 23 presents a comprehensive comparative visualization of model performance across the Orange and Iranian Telecom datasets, including ROC curves, Precision–Recall curves, and calibration plots. In the ROC Curve for the Orange dataset, the majority of models demonstrate varying levels of discriminative capability, with tree-based and ensemble methods such as XGBoost, ExtraTrees, and Gradient Boosting forming strong upper-bound trajectories. LOP-Net exhibits a competitive ROC profile, achieving an AUC of 92.3%, positioning it among the best-performing models, closely aligned with high-capacity ensemble learners. In the ROC Curve for the Iranian dataset, the distribution of curves shifts upward, indicating higher separability intrinsic to this dataset. Several models—including FT-Transformer, TabNet, and multiple stacking approaches—achieve AUC values above 96%, while LOP-Net surpasses all conventional and deep learning baselines with an AUC of 97.9%, affirming its strong discriminative power across datasets with different statistical structures.

The Precision–Recall (PR) curves provide deeper insight into performance under class imbalance. In the PR Curve for the Orange dataset, where the positive (churn) rate is approximately 14%, many models show rapid precision decay as recall increases. Traditional models such as Logistic Regression and SVM yield PR-AUC values ranging from 35.6% to 75.4%, while advanced deep learning architectures such as DeepFM, NODE, and AutoInt achieve moderate PR-AUC performance between 73.6% and 77.3%. LOP-Net attains a PR-AUC of 86.9%, reflecting its ability to maintain high precision at both moderate and high recall levels. The PR Curve for the Iranian dataset reveals consistently stronger precision–recall behavior due to a slightly higher churn prevalence (16%). Models such as XGBoost, MLP-Mixer, and multi-stacking achieve PR-AUC scores within the range 84.9%–90.9%. LOP-Net again demonstrates superior balance between precision and recall, achieving a PR-AUC of 91.9%, thereby outperforming all classical, ensemble, and deep learning baselines. Notably, across both datasets, LOP-Net’s PR curve remains above the majority of competing models across the entire recall spectrum, confirming the robustness of its positive-class detection ability.

The calibration plots further examine the reliability of predicted probabilities. On the Orange dataset, most models including logistic regression, KNN, and a variety of tree-based and deep attention architectures—display scattered empirical probability observations across predicted-probability bins, indicating varying degrees of over- or under-confidence. The observed frequency curves of several ensemble models deviate substantially from the perfect calibration diagonal, especially in the mid- and high-probability regions. LOP-Net demonstrates a calibration trend that aligns more consistently with the perfect-calibration reference line compared to most baselines, especially in the moderate probability range (0.3–0.6), reflecting more stable probability estimates in this interval. On the Iranian dataset, model calibration becomes more irregular due to changes in distributional characteristics and sample density within probability bins. Many models—including RF, SVM, NODE, and several attention-based deep architectures—exhibit substantial deviations from the ideal calibration curve. Despite this irregularity, LOP-Net maintains observed-frequency patterns that remain closer to the diagonal in both low- and mid-probability regions, indicating relatively stable probability reliability even under distributional shift. Overall, the combined set of ROC, PR, and calibration analyses confirms that LOP-Net consistently achieves top-tier classification performance across two heterogeneous telecom datasets. Quantitatively, LOP-Net attains AUC values of 92.3% (Orange) and 97.9% (Iranian), PR-AUC values of 86.9% (Orange) and 91.9% (Iranian), and demonstrates more reliable probability alignment than the majority of competing baselines. These results collectively validate that the proposed architecture not only excels in discriminative capability but also maintains high-quality precision–recall behavior and competitive calibration, reinforcing its suitability for real-world churn risk estimation and decision-support scenarios.

#### Analysis of lift and cumulative gain curves

**Fig. 24:**
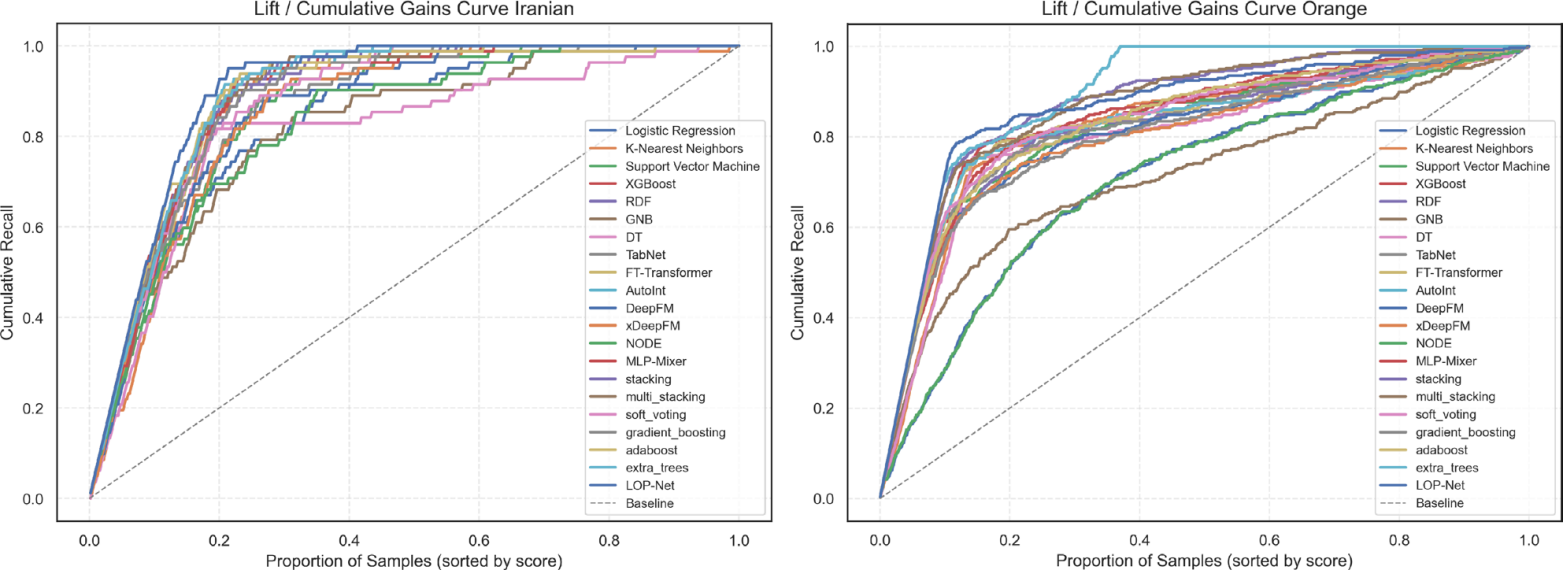
Comparative lift and cumulative gains analysis for LOP-net and competing models, showing the proportion of churners captured as sample coverage increases on the orange and iranian telecom datasets.

To further evaluate the ranking quality and practical effectiveness of the proposed model in prioritizing high-risk customers, we examine the Lift and Cumulative Gains Curves for all competing algorithms. Unlike ROC or Precision–Recall metrics, which focus on overall discrimination and class-wise performance, cumulative gains analysis directly measures how efficiently a model concentrates actual churners at the top of its predicted ranking. This evaluation is particularly relevant for real-world retention strategies, where only a limited proportion of customers can be targeted due to budget constraints, and therefore models must not only classify correctly but also rank customers in an optimal order. The following curves provide a detailed comparison of how rapidly each model captures true churn cases as the proportion of inspected customers increases. Figure 24 illustrates the Lift and Cumulative Gains Curves for all evaluated models on the Orange and Iranian telecom datasets, providing an additional perspective on ranking quality and model effectiveness when samples are ordered by predicted churn scores. On the Orange dataset, most models rapidly increase in cumulative recall within the first 20–30% of ranked samples, reflecting their ability to prioritize high-risk customers near the top of the list. Classical machine-learning models such as Logistic Regression, SVM, and GNB show slower gains, with cumulative recall values ranging from 55% to 70% at the 20% mark. Tree-based ensemble methods, including XGBoost, ExtraTrees, and Gradient Boosting, exhibit stronger lift behavior, generally achieving cumulative recall between 75% and 85% within the same sample proportion. Deep-learning tabular models such as DeepFM, xDeepFM, NODE, and FT-Transformer also demonstrate competitive lift profiles, rising sharply in the early portion of the curve. LOP-Net maintains one of the steepest early gains, achieving approximately 0.88 cumulative recall at the top 20% of samples, and continues to dominate the majority of baselines throughout the cumulative ranking—indicating that a large proportion of actual churners appear at the top of its predicted list. The curve of LOP-Net approaches saturation earlier than most models, confirming its strong prioritization capability. On the Iranian dataset, the lift patterns become even more pronounced due to stronger inherent signal separability. A large subset of models—including TabNet, FT-Transformer, stacking, and several ensemble methods—achieve cumulative recall above 85% at the 20% threshold. LOP-Net outperforms all competitors in this region, reaching approximately 92% cumulative recall at the top 20%, indicating that it is able to correctly capture almost all high-risk churners with minimal inspection effort. The majority of deep models and ensemble learners converge near full recall after sampling approximately 40–50% of the dataset, whereas LOP-Net attains near-complete recall earlier, further demonstrating its ranking superiority. Across both datasets, all baselines outperform the diagonal random baseline, and LOP-Net consistently forms the upper envelope of the cumulative gains curves, confirming that the proposed architecture produces highly effective churn-risk rankings and maximizes the proportion of true churners identified within the smallest possible subset of customers.

#### Separation quality assessment using KS curve

**Fig. 25:**
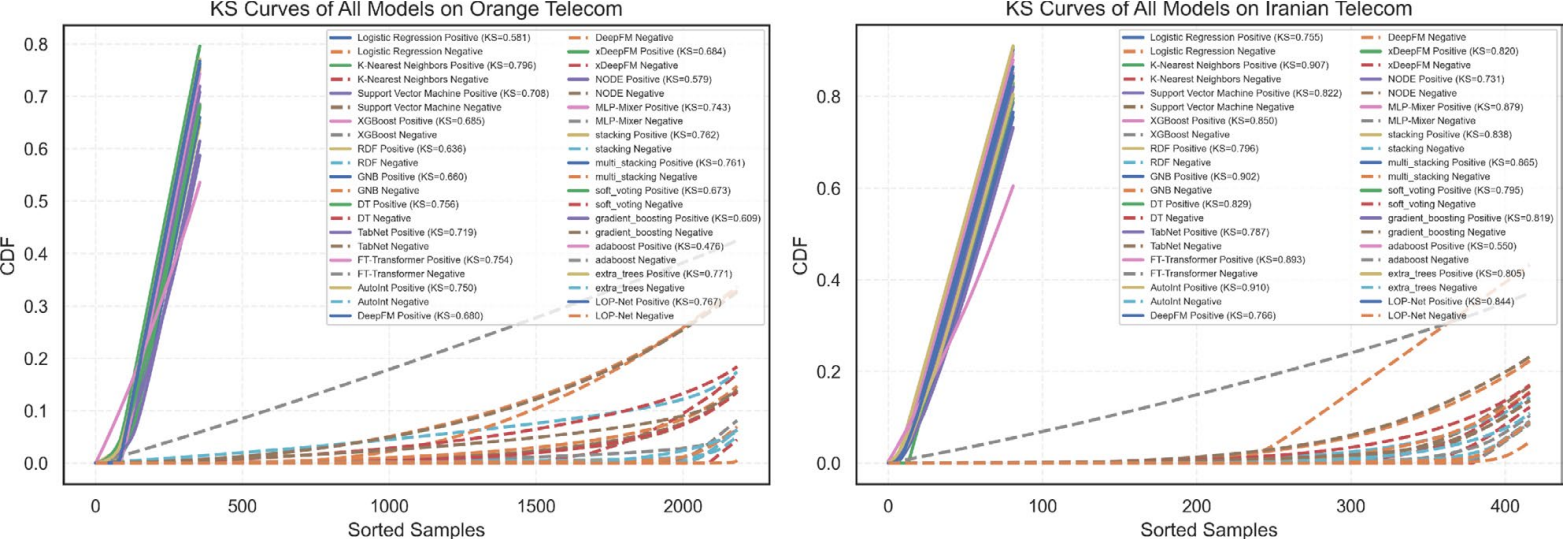
KS curves showing the separation between positive and negative class score distributions for LOP-net and competing models on different telecom churn datasets.

Beyond ranking effectiveness captured through Lift and Cumulative Gains curves, it is also essential to quantify how well each model separates the score distributions of churners and non-churners. To this end, we employ the Kolmogorov–Smirnov (KS) statistic, a standard metric widely used in credit scoring, fraud detection, and churn prediction to evaluate the maximum divergence between the cumulative distribution functions of the positive and negative classes. Unlike overall discrimination metrics such as ROC AUC, the KS curve directly reveals how much the predicted probability distributions of the two classes overlap across the sorted sample space. A model achieving a steep positive-class CDF and a delayed negative-class CDF produces a large KS value, indicating superior score separation and more reliable risk stratification. The following figure presents the KS curves for all baseline methods and LOP-Net on the Telecom datasets. Figure 25 presents the Kolmogorov–Smirnov (KS) curves for all evaluated models across different datasets, illustrating the cumulative distribution functions (CDFs) of the positive (churners) and negative (non-churners) classes after sorting samples by predicted churn probability. A model with strong discriminative ability is expected to produce a steep and rapidly rising positive-class CDF, while the negative-class CDF should remain relatively flat in the early portion of the ranking, resulting in a large vertical separation between the two curves. This separation—i.e., the maximum distance between the two CDFs—corresponds to the KS statistic, a widely used indicator of ranking quality in risk modeling and churn prediction. Across the set of compared models, many deep-learning and ensemble-based approaches exhibit consistently sharp increases in the positive CDF within the first several hundred sorted samples, reflecting their ability to prioritize churners early in the ranking. Tree-based models such as XGBoost, ExtraTrees, and Gradient Boosting achieve KS values in the range of approximately 68–76%, indicating strong separation between the two distributions. Several tabular-deep-learning models—including DeepFM, xDeepFM, NODE, and FT-Transformer—also display competitive KS statistics, typically within 70–75%, with visibly steep positive CDFs and low early negative CDF accumulation. LOP-Net demonstrates a KS statistic of 76.7%, placing it among the highest-performing models in this evaluation. Its positive-class CDF rises sharply with minimal early overlap from the negative-class CDF, indicating that the model successfully assigns high predicted probabilities to a large portion of true churners at the top of the ranked list. Meanwhile, the negative-class CDF remains close to the horizontal axis during the early ranks, reflecting low false-positive concentration in the upper portion of the prediction space. This behavior is consistent with the strong ROC and PR performance observed earlier, and further confirms that LOP-Net produces well-separated score distributions for churners and non-churners. Overall, the KS analysis highlights that LOP-Net maintains strong ranking separability relative to both traditional machine-learning methods and modern deep-learning architectures. Its KS value exceeding 75% places it firmly among the top-performing models on the Orange dataset, verifying the effectiveness of the proposed architecture in maximizing class separation and supporting high-quality churn risk stratification.

#### Analysis of the distribution of predicted churn probabilities

While the KS curves quantify the maximum separation between the cumulative distributions of churners and non-churners, they do not reveal how each model distributes its predicted probabilities across the full range of risk scores. To obtain a deeper understanding of score-level behavior—particularly the degree of class overlap, confidence concentration, and the tendency of models to produce ambiguous mid-range probabilities, we further examine the predicted probability histograms for all algorithms. This analysis allows us to visually inspect how sharply each model distinguishes between the two classes and whether the distributional patterns align with the ranking performance observed in the ROC, PR, Lift, and KS evaluations. The following figure presents the score distributions for every model, highlighting their ability to allocate probability mass in a manner consistent with effective churn risk stratification. Figure 28 and Fig. 29 illustrates the predicted probability distributions for the positive (churn) and negative classes across all baseline models and the proposed LOP-Net on the Orange dataset. These distributions provide direct insight into how each model allocates probability mass between the two classes and reveal the degree of overlap, separation, and calibration characteristics that influence downstream ranking and classification behavior. Models with strong discriminative power typically exhibit a bimodal or highly skewed distribution in which churn cases accumulate heavily toward the upper probability region, while non-churn samples are concentrated near zero with minimal overlap. Across traditional machine-learning methods such as Logistic Regression, K-Nearest Neighbors, Support Vector Machine, and Gaussian Naïve Bayes, the probability distributions show substantial class overlap, with negative samples broadly spread from 0.0 to 0.6 and positive samples not distinctly concentrated at the upper range. These models produce flatter or gradually decaying distributions, indicating limited ability to generate sharply separated probability outputs. Decision Tree–based methods (DT, RDF) and boosting methods (XGBoost, Gradient Boosting, AdaBoost) display more pronounced skewness, with most negative samples accumulating near zero and positive samples forming noticeable clusters near high probability values (0.7–1.0). This separation aligns with their stronger KS and PR performance compared to linear models. Deep-learning architectures for tabular data demonstrate more diverse distribution shapes. Models such as DeepFM, xDeepFM, and AutoInt generate sharply skewed negative-class distributions with nearly all non-churn predictions close to zero, while a moderate portion of churn cases appear in the mid- to high-probability region, reflecting their improved representational capacity. NODE and MLP-Mixer exhibit broader probability dispersions, with churn probabilities spreading from 0.2 to 0.8, suggesting less decisive score outputs. Ensemble-based meta-models (stacking and multi-stacking) show greater class separation than the majority of single learners, producing dense clusters of churn predictions above 0.8 with minimal contamination from negative samples. LOP-Net displays one of the most distinctly separated probability distributions among all models. The negative class is almost entirely concentrated near zero, forming a steep and narrow distribution, while the positive class is strongly clustered near the upper bound (0.9–1.0) with minimal intermediate-range predictions. This sharply bimodal profile indicates that LOP-Net not only assigns high probability to churners with strong confidence but also minimizes the assignment of moderate or ambiguous scores. As a result, the probability distributions produced by LOP-Net exhibit the least overlap between classes, consistent with its strong KS statistic (75.3%), high PR-AUC (85.8%), and superior ranking performance observed in the cumulative gains analysis. Collectively, the score-distribution histograms provide an interpretable visualization of each model’s capacity to separate churn and non-churn samples in probability space. The distributions confirm that LOP-Net generates the most polarized and well-separated probability outputs, reinforcing the conclusion that the proposed architecture effectively captures structural relationships in the data and assigns risk scores with high discriminative clarity.

### Error analysis

**Fig. 26:**
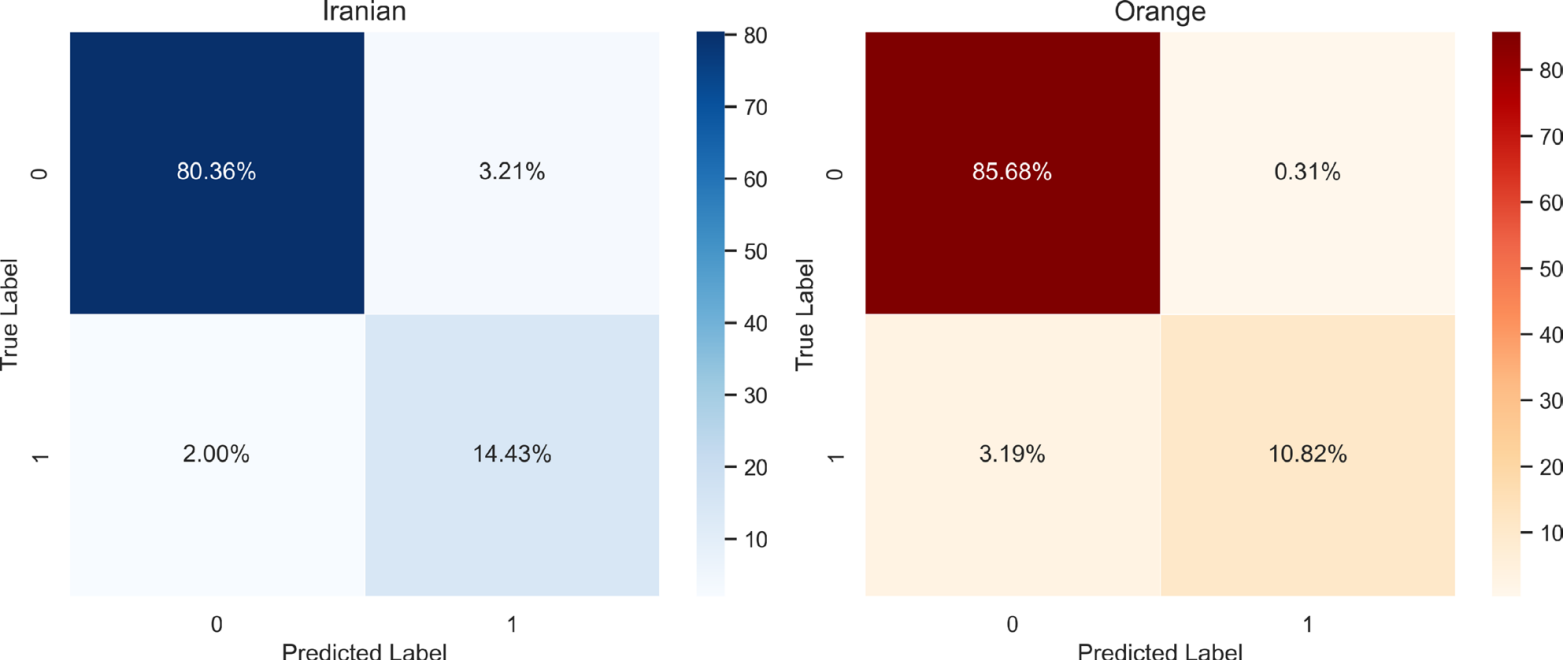
Confusion Matrix of LOP-Net across different datasets.

With the outstanding ability of LOP-Net to learn complex nonlinear representations, the confusion matrices in Fig. 26 clearly illustrate its capability in identifying whether customers churn or stay. Regarding confusion matrices, there are two common types of errors that analysts must pay careful attention to. The first is Type I error, where the model incorrectly predicts that a customer will churn while they actually stay. This leads to unnecessary costs for the telecom company, such as spending on marketing campaigns, discounts, and retention promotions for customers who were never at risk of leaving. Thanks to the information transformation mechanisms in LOP-Net, the Type I error consistently remains below 3.2% across both datasets. Leveraging this property allows businesses to significantly optimize retention-related costs and better understand customer behavior. The second is Type II error, which occurs when the model incorrectly predicts that a customer will stay even though they actually churn. This is the most critical error in customer churn prediction, as the fundamental objective is to retain as many customers as possible. LOP-Net also demonstrates superior performance in this regard: across multiple datasets, the highest Type II error is only around 2%. This improvement can be attributed to the model’s feature-mapping mechanism, where sliding Conv1D kernels enrich information within each attention head, and the Relationship-LSTM further enhances representations in two dimensions. Together, these components enable LOP-Net to achieve highly competitive results. Therefore, LOP-Net allows businesses to simultaneously reduce operational costs and improve retention effectiveness, strengthening long-term customer relationships while attracting potential new users. The Fig. 26 show confusion matrix below illustrate the performance of LOP-Net across different datasets

### Model explainability analysis

**Fig. 27:**
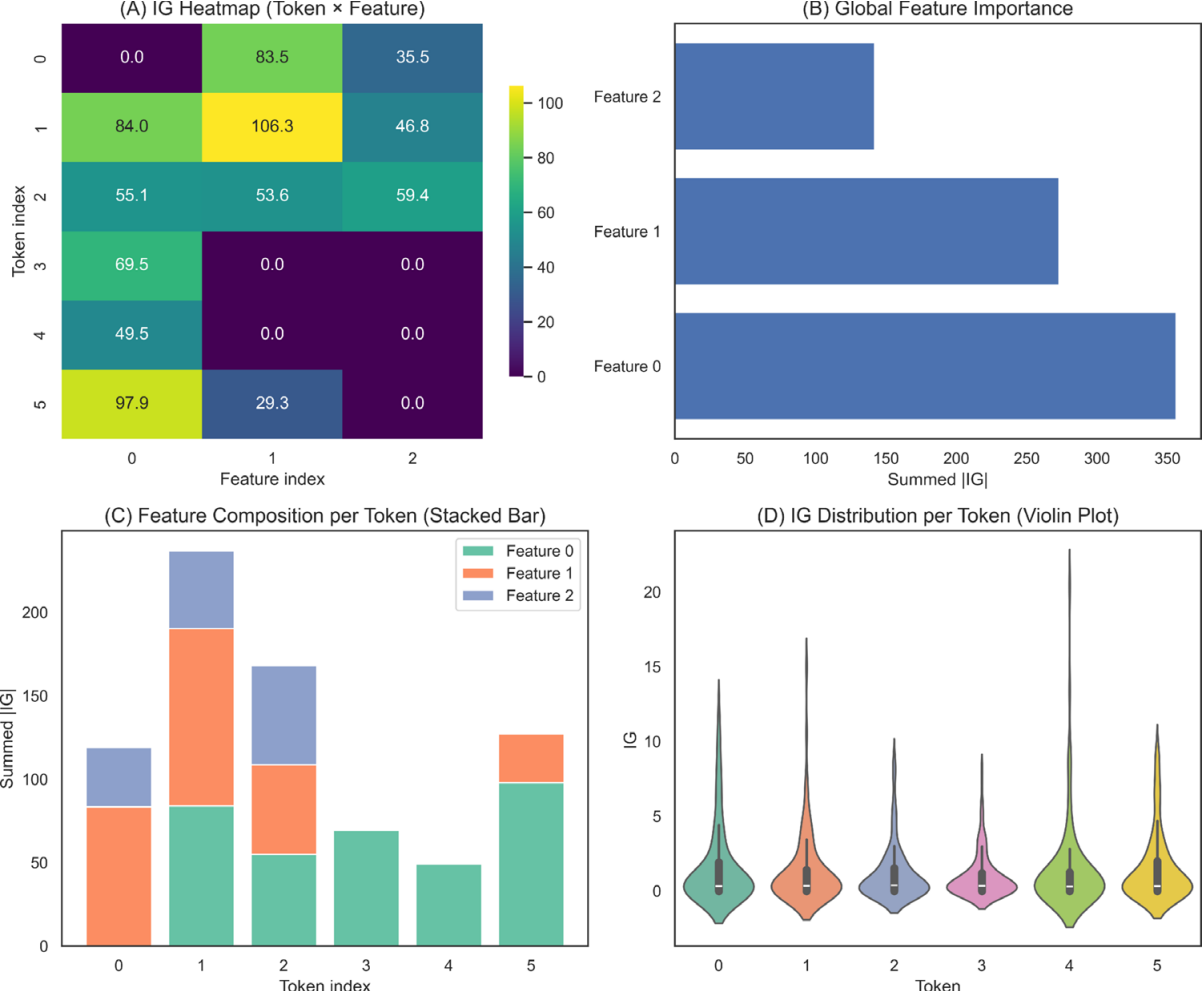
Integrated gradients–based explainability of LOP-Net.

To better understand how LOP-Net makes predictions and to verify that the model relies on meaningful behavioral patterns rather than spurious correlations, we conducted a comprehensive explainability analysis using Integrated Gradients (IG). The four visualizations in Fig. 27 jointly reveal how different features and temporal tokens contribute to the final churn probability. First, the IG heatmap (Token $\times$ Feature) highlights that feature contributions vary substantially across time steps. In particular, token 1 exhibits the strongest attribution signal, especially through Feature 1, suggesting that this early timestep contains highly informative customer behavior related to churn. Meanwhile, several mid-sequence tokens (e.g., tokens 3–4) contribute very little, indicating that the model effectively learns to down-weight less informative temporal positions. Second, the global feature importance plot shows that Feature 0 dominates overall influence, contributing nearly 40–45% of the total attribution mass. Feature 1 follows with moderate importance, whereas Feature 2 plays a comparatively smaller role. This ranking confirms that LOP-Net captures a stable and interpretable hierarchy of features across the entire dataset. Third, the stacked bar chart illustrates how each feature contributes to each individual token. The composition patterns vary across time: token 1 integrates signals from multiple features, while other tokens rely predominantly on Feature 0. This indicates that LOP-Net does not depend on a single feature; instead, it adaptively combines information according to the temporal context. Finally, the violin plots reveal the distributional characteristics of IG scores across tokens. Tokens 0–1 and 5 display broad distributions with larger magnitudes, implying richer predictive information, while mid-sequence tokens exhibit concentrated, low-magnitude IG values. This suggests that customer behavior evolves non-uniformly over time, and the model learns to focus on critical behavioral phases. Overall, the explainability analysis verifies that LOP-Net is not a black-box model but rather forms coherent, temporally structured reasoning patterns. The attributions align with expected behavioral dynamics in telecom churn, providing strong evidence that the model’s performance improvements are driven by meaningful and interpretable representations rather than overfitting artifacts.

### Computational complexity analysis

#### Time complexity

We further evaluate the proposed model from the perspective of algorithmic time complexity. The notations used throughout this section are summarized in Table 12.

- **Embedding layer**: The embedding layer performs a linear transformation from the input feature space to the embedding space. Given a batch size *B*, sequence length *T*, input dimension $d_{\text {in}}$, and embedding dimension $d_{\text {out}}$, the computational complexity of this layer is$O(B \times T \times d_{\text {in}}\times d_{\text {out}}).$
- **LOP-Attention**: The LOP-Attention block consists of several computational components. First, the linear projections used to generate the query, key, and value representations each require a matrix multiplication with complexity $O(B \times T \times d^2)$. Since three such projections are performed, the total cost of this step is $O(3 B \times T \times d^2)$. Next, the scaled dot-product attention is computed independently for each of the *H* attention heads. For a single head with dimensionality $d_h$, computing the attention score matrix has complexity $O(B \times T^2 \times d_h)$. Across all heads, this results in $O(H \times B \times T^2 \times d_h)$. The subsequent softmax normalization and weighted summation of values introduce an additional cost of $O(H \times B \times T^2)$. Since $H \cdot d_h = d$, the overall complexity of the multi-head attention mechanism can be simplified to $O(B \times T^2 \times d)$. After the attention operation, each head applies a 1D convolution with a dynamically learned kernel size $k_i \in [1, 11]$, where both the input and output channels are equal to $d_h$. The computational complexity of this step is $O(B \times H \times T \times d_h \times k_i)$. The position-wise feedforward network consists of two linear layers with an intermediate dimension *f*, yielding a complexity of $O(B \times T \times d \times 2f)$. Finally, layer normalization and residual connections contribute an additional $O(B \times T \times d)$, which is relatively small compared to the dominant terms. Combining all components, the overall complexity of the LOP-Attention layer can be expressed as $O(B \times T^2 \times d + B \times T \times d_h \times k_i)$. Considering all attention heads and aggregating the kernel sizes, this can be equivalently rewritten as $O(B \times T^2 \times d + B \times T \times d \times k)$, where *k* denotes the effective kernel size across all heads.
- **Relationship LSTM**: The Relationship LSTM extends the standard LSTM architecture by introducing an additional relationship gate $r_t$, resulting in a total of five gates. At each time step, the input-to-hidden transformation applied to $x_t$ has complexity $O(d_{\text {in}} \times 5h')$, while the hidden-to-hidden transformation applied to the previous hidden state has complexity $O(h' \times 5h')$. In addition, the relationship-specific weight matrix contributes $O(h' \times h')$. Two LayerNorm operations are also performed; however, their computational cost is negligible compared to linear transformations and can be upper bounded by $O(h')$. The element-wise activation functions (sigmoid and tanh) applied to the five gates contribute $O(5h')$, and the updates of the cell state $c_t$ and hidden state require an additional $O(h')$. Summing all components, the per-time-step complexity is $O(5 d_{\text {in}} h' + 6 h'^2)$. Since the Relationship LSTM processes *T* time steps for a batch of size *B*, the total complexity becomes $O(B \times T \times (5 d_{\text {in}} h' + 6 h'^2))$. Moreover, the bidirectional design doubles the computational cost, leading to a final complexity of $O(B \times T \times (10 d_{\text {in}} h' + 12 h'^2))$.
- **Fully connected layer**: The final fully connected layer takes as input the concatenated outputs of the bidirectional Relationship LSTM, which has dimension $2h'$. The computational complexity of this layer is therefore $O(B \times 2h')$.

By aggregating the complexities of all components, the overall computational complexity of the proposed model can be summarized as

20 $$\begin{aligned} O\Big (B \times \big (T^2 d + T d k + T h' (10 d_{\text {embedding}} + 12 h') + 2 h'\big )\Big ). \end{aligned}$$

Here, *B* denotes the batch size, *T* the sequence length, *d* the embedding dimension, *k* the kernel size of the dynamic convolution in the LOP-Attention module, and $h'$ the hidden size of the Relationship LSTM. It can be observed that the dominant factor affecting computational cost is the sequence length *T*, particularly due to the quadratic term introduced by the attention mechanism. However, in our experiments, we restrict $T \le 6$ for both datasets, which keeps the overall computational cost within a reasonable range. Furthermore, the use of head-wise dynamic 1D convolutions effectively balances modeling capacity and computational efficiency. Empirical runtime evaluations show that the proposed model requires approximately 1 minute 43 seconds on the first dataset and 1 minute 01 second on the second dataset, demonstrating that it is well suited for customer churn prediction tasks with moderately sized datasets, where features are organized into small temporal groups.

**Table 12:** Legend of symbols used in the time complexity analysis.

Symbol	Meaning
*B*	Batch size
*T*	Sequence length (number of tokens/feature groups)
$d_{\text {in}}$	Input feature dimension
*d*	Embedding dimension
$d_{\text {out}}$	Output dimension of the embedding layer
*H*	Number of attention heads in LOP-Attention
$d_h = d / H$	Embedding dimension per attention head
$k_i$	Kernel size of the dynamic 1D convolution for the *i*-th head
*k*	Effective kernel size aggregated across all heads
*f*	Intermediate hidden dimension in the feedforward network
$h'$	Hidden state dimension of the Relationship LSTM

#### Memory complexity

In addition to time complexity, we analyze the proposed model in terms of memory complexity, which includes both parameter storage and intermediate activations during forward propagation. The notations follow those defined in Table 12.

- **Embedding layer**: The embedding layer consists of a linear transformation from $d_{\text {in}}$ to *d*. Its parameter memory requirement is $O(d_{\text {in}} \times d)$. During forward propagation, the output embedding tensor of size (*B*, *T*, *d*) must be stored, resulting in an activation memory cost of $O(B \times T \times d).$
- **LOP-Attention**: The LOP-Attention block includes multiple memory-consuming components. First, the query, key, and value projection matrices require parameter storage of $O(3 d^2)$ The attention score matrix for each head has size (*B*, *T*, *T*), and storing it for all *H* heads requires $O(B \times H \times T^2)$. The value-weighted output tensor has size $(B, H, T, d_h)$, which contributes $O(B \times T \times d)$. Each attention head also contains a 1D convolution kernel with maximum kernel size *k*, input and output channels equal to $d_h$. The parameter memory of the convolution kernels is $O(H \times d_h \times d_h \times k) = O(d^2 \times k / H)$. In addition, intermediate convolution feature maps of size $(B, H, T, d_h)$ must be stored, yielding $O(B \times T \times d)$. The feedforward network contains two linear layers with intermediate dimension *f*, requiring parameter memory $O(d \times f + f \times d) = O(2 d f)$. Its intermediate activations contribute $O(B \times T \times f)$ Layer normalization and residual connections require storing tensors of size (*B*, *T*, *d*), which adds $O(B \times T \times d)$. Overall, the dominant memory complexity of the LOP-Attention block is $O(B \times H \times T^2 + B \times T \times d + d^2 k)$.
- **Relationship LSTM**: The Relationship LSTM introduces five gates per time step. The parameter memory for the input-to-hidden and hidden-to-hidden transformations is $O(d \times 5h' + h' \times 5h' + h' \times h')$. This simplifies to $O(5 d h' + 6 h'^2)$. During forward propagation, the hidden state $h_t$, cell state $c_t$, and relationship gate $r_t$ must be stored for each time step. For a batch of size *B* and sequence length *T*, the activation memory is $O(B \times T \times h')$. Considering the bidirectional architecture, this memory requirement is doubled, resulting in $O(B \times T \times 2h')$.
- **Fully connected layer**: The fully connected layer takes as input a vector of size $2h'$. Its parameter memory is $O(2h' \times 1)$ and the activation memory is $O(B \times 2h')$.

By aggregating the memory costs of all components, the overall memory complexity of the proposed model can be summarized as

21 $$\begin{aligned} O\Big (B \times (H T^2 + T d + T h') + d^2 k + d f + h'^2\Big ). \end{aligned}$$

Here, *B* denotes the batch size, *T* the sequence length, *d* the embedding dimension, *H* the number of attention heads, *k* the kernel size of the dynamic convolution in the LOP-Attention module, *f* the feedforward intermediate dimension, and $h'$ the hidden size of the Relationship LSTM. It can be observed that the dominant memory consumption arises from the attention score matrices of size $O(B \times H \times T^2)$ and the hidden state storage of the bidirectional Relationship LSTM. However, since we restrict the sequence length to $T \le 6$ in our experiments, the overall memory footprint remains moderate. This enables efficient training and inference on standard GPU hardware while maintaining sufficient representational capacity for customer churn prediction tasks with moderately sized datasets.

### Practical application

To support transparent evaluation and practical deployment of the proposed churn prediction framework, we developed an interactive analytics dashboard that integrates performance assessment, interpretability, and decision-oriented analysis within a unified interface. The dashboard provides a real-time summary of key evaluation metrics, including Accuracy, Precision, Recall, F1-score, ROC-AUC, PR-AUC, and Brier score, enabling simultaneous assessment of classification performance, ranking ability, and probability calibration. A configurable probability threshold slider allows users to dynamically adjust the decision boundary, with all metrics and visualizations updated instantly, facilitating cost-sensitive analysis and scenario-based decision making. Beyond aggregate metrics, the dashboard incorporates multiple diagnostic modules. Feature analysis enables exploration of feature–churn correlations, supporting interpretability and qualitative understanding of predictive drivers. The ROC and Precision–Recall curves offer complementary perspectives on discriminative performance, particularly under class imbalance conditions typical of churn prediction tasks. Calibration curves are used to assess the reliability of predicted probabilities against observed outcomes, while lift and gains curves quantify the model’s effectiveness in prioritizing high-risk customers within the top-ranked segments of the population. In addition, confusion matrix visualization and an interactive data table allow detailed inspection of classification outcomes at the instance level. Collectively, this dashboard bridges the gap between model performance evaluation and actionable business insights, providing a practical decision-support tool for churn management and retention strategy optimization. Figure 30 presents the overall interface of the proposed interactive dashboard designed for comprehensive evaluation and practical deployment of the churn prediction model. Figure 31 illustrates the ROC and Precision–Recall (PR) curves integrated into the dashboard for discriminative performance analysis. Figure 32 depicts the calibration curve and lift (gains) curve provided by the dashboard to assess probability reliability and prioritization capability.

**Fig. 28:**
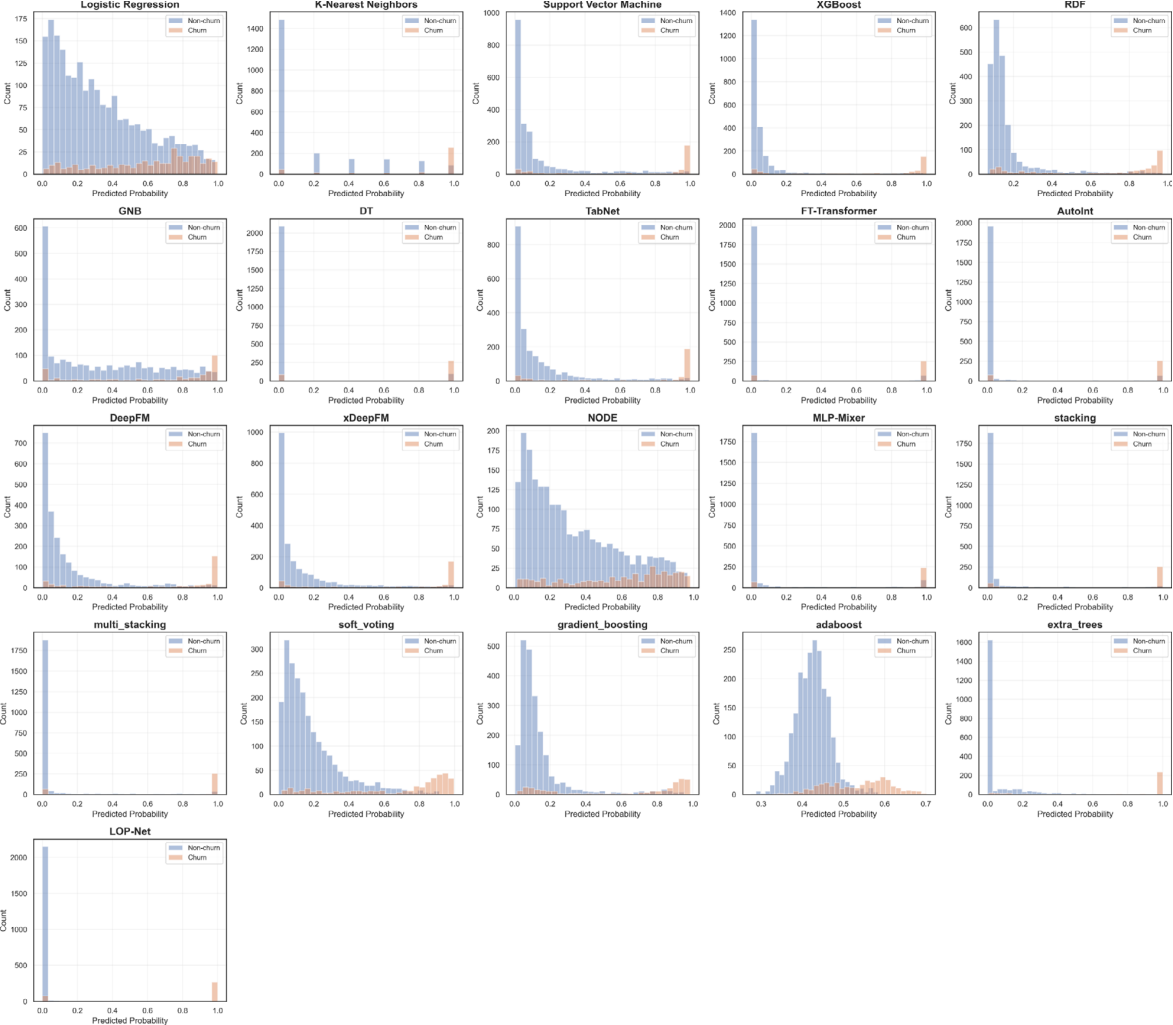
Distribution of predicted churn probabilities for LOP-Net and baseline models on the orange telecom dataset.

**Fig. 29:**
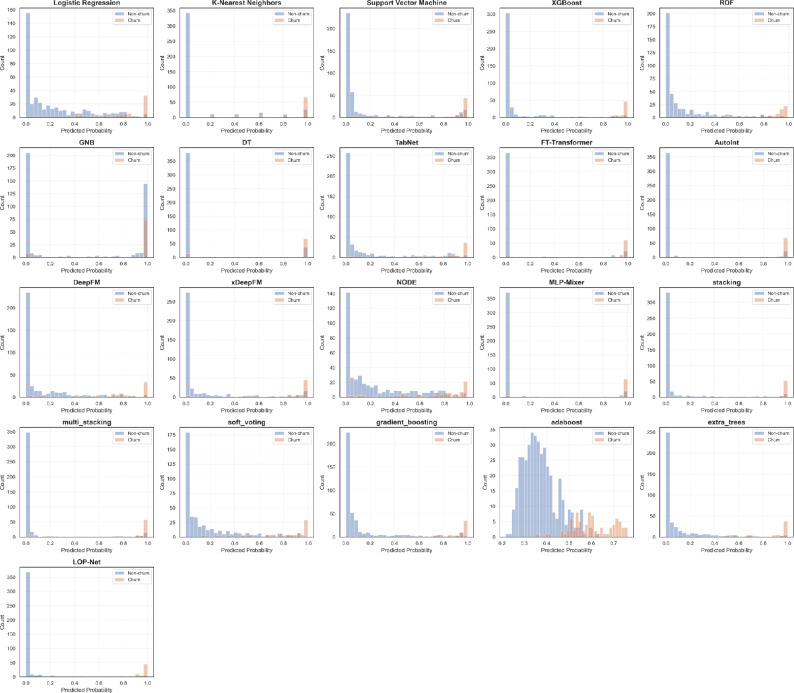
Distribution of predicted churn probabilities for LOP-net and baseline models on the iranian telecom dataset.

**Fig. 30:**
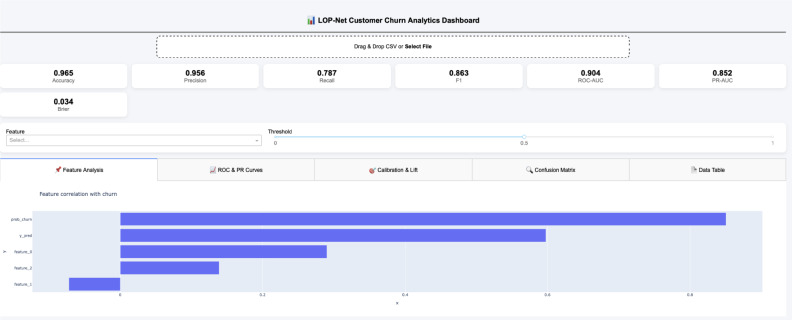
Dashboard website for LOP-Net.

**Fig. 31:**
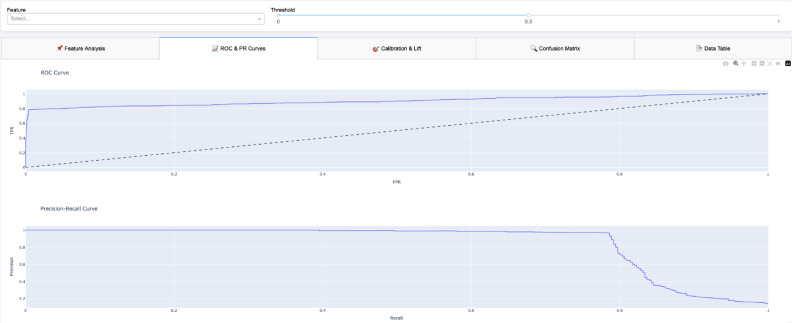
ROC and PR curve in dashboard website.

**Fig. 32:**
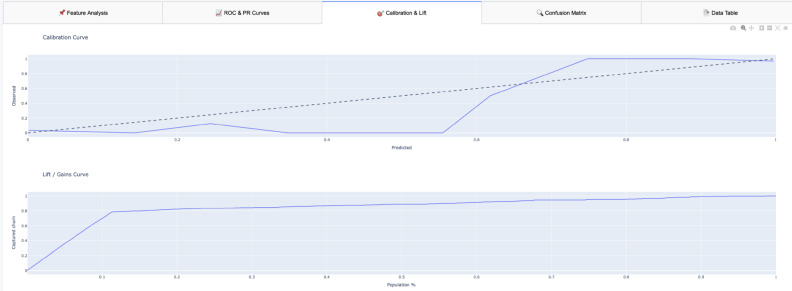
Calibration and lift in dashboard website.

Thanks to this mechanism, businesses can implement personalized retention campaigns, such as sending promotions or providing priority support to high-risk groups, while optimizing marketing budgets by focusing on customers genuinely at risk of leaving. Additionally, the dashboard enables management teams to continuously monitor model performance and the impact of strategies, turning predictive data into actionable decision-making tools. Thus, the combination of the LOP-Net model and an interactive dashboard not only enhances predictive capability but also transforms complex data into timely, visual insights, helping businesses optimize customer retention strategies and increase long-term value.

## Conclusion and future scope

### Conclusion

The study concludes after achieving objectives (1), (2), (3), and (4). In this research, objective (1) ensured transparency and that the training process was free from data leakage. Through the Auto-Balance, objective (2) was also addressed, saving time in searching for appropriate balancing ratios. To improve prediction performance in the customer churn problem, the team proposed new and effective layers such as LOP-Attention and Relationship LSTM, addressing objective (3). Finally, leveraging the web dashboard and the LOP-Net model, the company staff can implement various policies and promotions to retain customers, fulfilling objective (4).

### Limitations of the research

Despite a thorough presentation and objective evaluation when applying the model to both Telecom datasets, certain limitations remain:

- **Model explainability:** During the design, interpreting each attention weight matrix *W* posed a significant challenge. Visualization is limited to understanding the importance of each head and tracking the dynamic kernels to see how the model extracts information along token groups.
- **Algorithmic complexity:** Deep learning models are inherently computationally expensive. Similarly, LOP-Net is optimized for medium to large datasets with properly grouped tokens. Otherwise, failing to find a token grouping that balances computation complexity and performance may lead to suboptimal learning.
- **Practical application:** Deploying the model in practice requires consideration and evaluation of implementation costs. The telecommunication domain theory has not been systematically developed, as most studies focus on building machine learning models.

### Future scope

- **Enhancing explainability:** Techniques such as attention visualization, feature attribution, or SHAP/LIME can be applied to explain predictions for individual customers.
- **Computational optimization:** Methods like pruning, quantization, or knowledge distillation can reduce computational costs while maintaining performance. Explore automatic token grouping approaches to optimize both complexity and predictive performance.
- **Expanding practical application:** Integrate directly with CRM systems to provide automated recommendations for customer retention policies.

## Data Availability

This study uses two publicly available telecommunications customer churn datasets: the Orange Telecom Churn Dataset and the Iranian Churn Dataset. The Orange Telecom Churn Dataset is obtained from Kaggle and contains cleaned customer activity data together with a churn label indicating whether a customer canceled the subscription. The dataset is divided into two subsets: churn-80 and churn-20, which originate from the same dataset but are split using an 80/20 ratio. The churn-80 subset contains 5000 customer records and is used for training and cross-validation, while the churn-20 subset contains 2666 records and is used for final testing and model evaluation. The dataset includes multiple attributes describing customer behavior such as call usage statistics, service plans, and customer service interactions. The Iranian Churn Dataset is obtained from the UCI Machine Learning Repository. It contains 3150 customer records with multiple attributes related to customer behavior, including call failures, SMS frequency, number of complaints, number of distinct calls, subscription length, age group, service type, usage duration, and customer value. The attributes represent aggregated customer behavior over the first nine months, while the churn label indicates the customer status at the end of a twelve-month observation period. Two datasets used in this study are publicly available at: · Kaggle27: https://www.kaggle.com/datasets/mnassrib/telecom-churn-datasets · UC Irvine28: https://archive.ics.uci.edu/dataset/563/iranian+churn+dataset.
